# Molecular Epidemiology of Pityriasis Versicolor Reveals a Remarkably High Prevalence of *Malassezia japonica* in Coastal South India

**DOI:** 10.3390/microorganisms14102318

**Published:** 2026-10-12

**Authors:** Jayaprakash Sasikumar, Bharati Naik, Manjunath M. Shenoy, Tamalika Manna, Shankar Prasad Das

**Affiliations:** Cell Biology and Molecular Genetics, Yenepoya Research Centre (YRC), Yenepoya (Deemed to be University), Mangalore 575018, India; jeyaprakash6397@gmail.com (J.S.); manjunath576117@gmail.com (M.M.S.); tama2000manna@gmail.com (T.M.)

**Keywords:** *Malassezia*, pityriasis versicolor, epidemiology, prevalence, skin fungi

## Abstract

Pityriasis versicolor (PV) is a common superficial mycosis caused by lipophilic yeasts of the genus *Malassezia*. Despite its high prevalence, comprehensive molecular epidemiological data on *Malassezia* species associated with PV remain limited in several regions of India. Therefore, our study investigated the molecular epidemiology, demographic characteristics, seasonal variation, clinical manifestations, anatomical distribution, and associated predisposing factors among patients with PV from the coastal region of Karnataka. A total of 147 patients clinically diagnosed with PV were included in this study. Skin scrapings were isolated and cultured on lipid-rich media, and isolates were identified using polymerase chain reaction–restriction fragment length polymorphism (PCR-RFLP) and internal transcribed spacer (ITS) sequencing. Molecular identification was performed for 182 *Malassezia* isolates. *Malassezia furfur* was the predominant species at 51.1% (93 isolates), followed by *Malassezia japonica* at 34.1% (62), *Malassezia restricta* at 5.5% (10), *Malassezia slooffiae* at 3.3% (6), *Malassezia sympodialis* at 2.2% (4), *Malassezia arunalokei* at 2.2% (4), *Malassezia globosa* at 1.1% (2), and *Malassezia pachydermatis* at 0.6% (1). In addition, 20 non-*Malassezia* yeast isolates were identified, including *Candida*, *Rhodotorula*, *Meyerozyma*, and *Trichosporon*. The study population had a mean age of 27.8 ± 14.9 years, with a male-to-female ratio of 2.2:1. Most cases occurred among individuals aged 10–39 years, while *M. furfur* remained the predominant species across all age groups and both genders. Seasonal variation was evident, with the highest number of cases recorded during September. Clinically, hypopigmented macules were the predominant lesion type, and the back was the most frequently affected anatomical site. Underlying medical conditions or potential predisposing factors were identified in 18 patients (12.2%), with alcohol-related disorders and immunosuppressive conditions being the most frequently documented. Analyzing all these data, the present study provides updated molecular epidemiological data on *Malassezia* species causing PV in the coastal region of Karnataka. The predominance of *M. furfur* and the unexpectedly high prevalence of *M. japonica* highlight the importance of molecular identification for accurate species-level characterization.

## 1. Introduction

The skin is the body’s largest organ and a complex ecological landscape that supports a rich and dynamic microbial community [1]. Within this ecosystem, fungi constitute an important and often underappreciated component; their interactions with host tissues and with other members of the microbiome influence both health and disease [2,3]. Among cutaneous fungi, species of the genus *Malassezia* are unique in their lipid dependence and are closely associated with the stratum corneum and pilosebaceous units [4]. Although commonly regarded as commensals, *Malassezia* spp. are also implicated in a spectrum of dermatological conditions ranging from superficial pigmentary disorders to inflammatory dermatoses such as pityriasis versicolor (PV), seborrheic dermatitis (SD), atopic dermatitis (AD), and Malassezia folliculitis (MF) [5]. Among those, pityriasis versicolor (PV) is a classic example of a superficial mycosis that presents as a persistent, recurrent infection characterized by fine scaling patches of hypo- or hyperpigmentation. While the condition is relatively harmless, there are various types of pityriasis versicolor, which include inverse, atrophic, folliculocentric, papular, confetti, hyperkeratotic, annular, and extensive forms [6,7]. Despite its benign nature, pityriasis versicolor can severely affect the quality of life and psychological state of patients due to its persistence, cosmetic appearance, and repeated occurrence [8,9]. Given their high prevalence in tropical and subtropical areas, understanding the epidemiology and ecology of *Malassezia* species is necessary for a better understanding of the organisms involved, and their ecology on human skin has clear clinical and public health relevance [10].

The biology of *Malassezia* underlies both its commensal and its pathogenic potential. These yeasts are obligate or highly lipophilic, lacking the ability to synthesize long-chain fatty acids de novo and therefore relying on exogenous lipids available in sebum and other skin surface lipids [4]. This metabolic specialization explains their preference for sebaceous sites and their sensitivity to microenvironmental factors such as humidity, temperature, and sebum composition. In addition to nutritional dependence, *Malassezia* spp. exhibit considerable phenotypic and genetic diversity, with species differing in enzyme repertoires (lipases and phospholipases) [11,12,13], in the production of secondary metabolites (such as azelaic acid) [14] that can affect melanogenesis or provoke inflammation, and in susceptibility to antifungal agents [15,16]. These differences are clinically meaningful because species-level variation may influence lesion morphology, pigmentary outcomes, and response to therapy. Despite this, routine clinical practice often treats PV empirically without species-level identification, partly due to a historical reliance on morphological and culture-based methods that offer limited resolution.

Epidemiological studies have demonstrated that *Malassezia* species distributions are not uniform globally [17,18,19,20,21,22], and even within countries [23,24,25,26,27,28,29,30]. Climatic variables, host demographics, cultural practices, and methodological choices in detection all interact to shape observed prevalence patterns. Tropical regions with high humidity and temperature frequently report distinct species profiles compared with temperate zones [31]; similarly, urban versus rural settings [32], age distributions [33], and hygiene behaviors [34,35] can alter the local skin habitat and thus the distribution and dynamics of the fungal community. However, a major limitation of previous studies is their dependence on conventional culture-based methods and biochemical assays for species identification. Conventional culture methods often fail to recover fastidious organisms such as *Malassezia*, which have slow growth rates and require lipid-enriched media and specialized culture conditions. At the same time, biochemical assays have limited discriminatory power for the accurate identification of closely related and newly identified species [4,36]. This limitation has historically led to an underestimation of species diversity. In recent years, the advent of molecular techniques has significantly improved the resolution and accuracy of fungal identification. Tools such as PCR-based assays targeting the ITS region, PCR-RFLP, sequencing of ribosomal DNA (ITS, D1/D2 domains), species-specific multiplex PCR, and, more recently, next-generation sequencing (NGS) and metagenomic approaches have made it possible to detect even low-abundance and slow-growing species with high precision [37,38]. These techniques overcome many of the limitations associated with culturing by providing culture-independent identification, thereby capturing a more complete picture of the cutaneous mycobiome. Consequently, regional epidemiological surveys that integrate robust clinical sampling with both culture-based and molecular approaches provide the most reliable and comparable data on species composition across studies, enabling a deeper understanding of geographic variability and its clinical implications.

Despite a growing literature on *Malassezia* epidemiology, substantial regional gaps remain, particularly in many tropical coastal zones with distinct climatic and socio-cultural characteristics. Local studies are therefore essential to inform practice: they establish baseline prevalence, reveal dominant and emerging species, and provide isolates for functional and genomic study. Such work is particularly valuable when it bridges clinical sampling with laboratory characterization, culture, and molecular typing. These multi-layered datasets enable robust inference about which species are incidental colonizers and which are likely contributors to pathogenesis, and they set the stage for prospective correlation with treatment outcomes. Therefore, this study was designed to provide a rigorously collected, region-specific profile of *Malassezia* species associated with PV, combining classical mycological and molecular methods to move beyond descriptive taxonomy toward a clinically relevant understanding of *Malassezia* prevalence in a tropical coastal population.

## 2. Materials and Methods

### 2.1. Study Design and Patient Recruitment

This prospective, cross-sectional, single-center observational study was carried out at the Department of Dermatology and the Yenepoya Research Centre, Yenepoya (Deemed to be University), Mangalore, between January 2021 and April 2026. The study was approved by the Yenepoya Ethics Committee (Approval No. YEC-1/2019/018 and YEC-1/2023/351). All patients aged 5 to 80 years, of both sexes, with clinical manifestations indicative of pityriasis versicolor (PV) and a positive potassium hydroxide (KOH) mount on direct microscopy were included in this study after obtaining their written informed consent (Figure 1). Some of the common differential diagnoses, such as pityriasis alba, early-stage vitiligo, and post-inflammatory hypopigmentation, were ruled out through appropriate clinical and laboratory testing. The diagnosis of pityriasis versicolor was made based on clinical presentation, supplemented with dermoscopy and a positive KOH mount. Under direct microscopic examination, a “spaghetti and meatballs” pattern containing round to oval yeast cells with short and curved hyphae was observed in all cases. Relevant demographic and clinical data were recorded on a standard case record form at the time of recruitment. Following sample collection, patients were managed in accordance with standard dermatological practice. Localized and mild cases were treated with topical antifungal agents, including 2% ketoconazole cream and 2.5% selenium sulfide lotion. Patients with extensive, recurrent, or treatment-resistant disease were considered for systemic antifungal therapy, primarily oral fluconazole or itraconazole, based on the severity of infection and extent of skin involvement, and were informed about the recurrent nature of the disease and the importance of adherence to treatment and follow-up.

### 2.2. Sample Collection and Primary Isolation

Skin scrapings were obtained from the active edge of lesions by a dermatologist using a sterile glass slide. Scrapings were transferred to sterile, folded black cardboard sheets, labelled, and delivered to the laboratory on the same day. Each specimen was inoculated onto modified Dixon’s agar (mDA (HiMedia Laboratories, Thane, India)) and modified Leeming and Notman agar (mLNA (HiMedia Laboratories, Thane, India)) media supplemented with chloramphenicol (25 mg/mL) to suppress bacterial overgrowth, following standard laboratory practices [5]. Plates were incubated under conditions favorable for lipophilic yeasts (approximately 30–32 °C) and monitored daily for growth; they were observed for 5–10 days to allow recovery of slow-growing colonies (Figure 2A). Colony morphology (texture, color, and size) was recorded. Distinct colonies were subcultured by streaking to obtain single colonies for isolation (Figure 2B).

### 2.3. DNA Extraction and PCR Amplification

Genomic DNA was extracted from freshly grown pure colonies using the fungal DNA isolation kit according to the manufacturer’s instructions (Norgen Biotek, Thorold, ON, Canada). DNA concentration and purity were assessed using a Colibri microvolume spectrophotometer (Berthold Technologies, Bad Wildbad, Germany), and aliquots were stored at −20 °C until further use. The ITS region of rDNA was amplified for species identification using the primers ITS3 (5′-GCATCGATGAAGAACGCAGC-3′) and ITS4 (5′-GCATATCAATAAGCGGAGGA-3′). PCR reactions were assembled using Taq Master Mix (HiMedia Laboratories, Thane, India) with template DNA, primers, and nuclease-free water in a final reaction volume as per the manufacturer’s recommendations. Thermal cycling conditions consisted of initial denaturation at 95 °C for 3 min; 40 cycles of denaturation at 95 °C for 1 min, annealing at 52 °C for 1 min, and extension at 72 °C for 1 min; followed by a final extension at 72 °C for 10 min. Amplicons were separated on 1.5% agarose gels and visualized under UV illumination using a Gel Doc system (Vilber, Collégien, France).

### 2.4. RFLP Analysis and Sequencing

Species differentiation was performed by restriction fragment length polymorphism (RFLP) analysis of ITS PCR products. Digestion was carried out with *Alu* I (New England Biolabs, Ipswich, MA, USA) at 37 °C for 4–5 h following the manufacturer’s protocol. Digested fragments were resolved on 1.5% agarose gels, and banding patterns were compared with reference RFLP profiles of standard *Malassezia* species to assign species identity (Figure 2C). Isolates that produced ambiguous or non-diagnostic RFLP patterns were selected for Sanger sequencing confirmation. For sequencing, the ITS region (ITS1–ITS4) was amplified, and single-pass Sanger sequencing was performed. Sequence reads were quality-checked and queried against the NCBI database using Nucleotide BLAST (V 2.15.0) for species confirmation; final species assignments were made based on the best BLAST hit(s) and alignment quality.

### 2.5. Multiple Sequence Alignment and Phylogenetic Analysis

Representative nucleotide sequences were aligned using Clustal Omega (V 1.2) with the sequence type set to DNA and the default alignment parameters. The resulting multiple sequence alignment was exported in FASTA alignment format and imported into MEGA (version 11.0.3) for subsequent phylogenetic analyses. The best-fit nucleotide substitution model was determined using the Find Best DNA/Protein Models (Maximum Likelihood) function. The Jukes–Cantor (1969) model with gamma-distributed rate variation among sites (JC + G) was selected as the optimal model because it exhibited the lowest Bayesian information criterion (BIC) score among the models evaluated. Phylogenetic relationships were inferred using the maximum likelihood (ML) method implemented in MEGA under the JC + G substitution model. Evolutionary rate heterogeneity among sites was modeled using a discrete gamma distribution. The initial tree for the heuristic search was generated automatically using the neighbor-joining method, followed by tree optimization using the nearest-neighbor interchange (NNI) algorithm. The robustness of the inferred phylogeny was assessed by 100 bootstrap replicates. The final phylogenetic tree was visualized and exported using MEGA for downstream analysis.

## 3. Results

### 3.1. Demographic Characteristics of the Study Population

A total of 147 patients clinically diagnosed with PV were included in the study. Of these, 68.7% (101) were males, and 31.3% (46) were females, corresponding to a male-to-female ratio of approximately 2.2:1. The predominance of male patients indicates a higher occurrence of PV among males in the study population. The age of the participants ranged from 5 to 80 years, with a mean age of 27.8 ± 14.9 years. The highest proportion of patients was observed in the 20–29-year-old age group, accounting for 29.9% (44) of cases, followed closely by the 10–19-year-old age group with 28.6% (42) of cases. Together, these two age groups represented 58.5% of the total study population, indicating that PV was most frequently encountered during adolescence and early adulthood. Patients aged 30–39 years accounted for 17.7% (26) of cases, while those aged 40–49 years and 50–59 years accounted for 8.8% (13) and 7.5% (11) of cases, respectively. The prevalence of PV declined markedly in the older age groups, with only 0.7% (1) patient in the 60–69-year-old age category and 2.0% (3) patients in the 70–80-year-old age category. Similarly, children aged 0–9 years represented a relatively small proportion of the study population, comprising 4.8% (7) of cases. Overall, individuals aged 10 to 39 years accounted for 76.2% (112) of the 147 patients, indicating that PV predominantly affected adolescents and young adults in the present study (Supplementary Table S1), with active sebaceous glands and sebum secretion that favor the growth of *Malassezia*.

### 3.2. Hypopigmented Macules Were the Most Common Clinical Presentation of PV

The clinical manifestations of PV exhibited considerable variation in terms of lesion pigmentation and morphology. A total of 167 clinical lesion presentations were documented among the study population, encompassing macules, plaques, papules, patches, and nonspecific lesions with hypopigmented, hyperpigmented, or erythematous characteristics (Supplementary Table S2). Hypopigmented macules represented the most frequently observed clinical presentation, accounting for 46.1% (77) of all recorded lesions. This was followed by hyperpigmented macules, which accounted for 21% (35) of lesions, making them the second most common manifestation. Together, hypopigmented and hyperpigmented macules accounted for 67.1% (112) of all clinical presentations, indicating that macular lesions were the predominant morphological feature of PV in the study population.

Among the plaque-type lesions, erythematous plaques were the most common, accounting for 6% (10) of cases, followed by hyperpigmented plaques at 4.2% (7) and hypopigmented plaques at 2.4% (4). Hypopigmented patches were observed in 3.6% (6) of patients, and erythematous macules in 3.6% (6) of patients. Papular lesions were comparatively uncommon, with erythematous papules accounting for 3.0% (5), hyperpigmented papules for 2.4% (4), and hypopigmented papules for 1.8% (3) of the recorded presentations. A small number of patients presented with nonspecific descriptions of lesions. Hypopigmented lesions without further morphological characterization were documented in 1.8% (3) of cases, whereas only one (0.6%) patient exhibited a nonspecific hyperpigmented lesion, representing the least frequently recorded clinical presentation. When the lesions were grouped according to pigmentation, hypopigmented manifestations predominated, comprising 53.9% (90) of the 167 recorded lesions, including hypopigmented macules, patches, plaques, papules, and nonspecific hypopigmented lesions. In comparison, hyperpigmented lesions accounted for 28.1% (47) of lesions, while erythematous lesions comprised 12.6% (21) of lesions (Supplementary Table S2). The remaining presentations represented mixed or less frequently encountered lesion types. This indicates that alterations in skin pigmentation, particularly hypopigmented macules, constituted the principal clinical manifestation of PV in the study population. Notably, the study identified several atypical clinical presentations of PV, demonstrating a broader clinical spectrum beyond its conventional macular presentation. A facial presentation of PV was observed in 28 patients, making it the most frequently encountered atypical presentation in the study population. Folliculocentric PV was identified in 18 patients, characterized by lesions centered on hair follicles, whereas inverse PV was observed in 9 patients, involving atypical intertriginous sites. Hyperkeratotic PV was documented in 6 patients, representing another less commonly described morphological variant. The occurrence of these atypical forms, particularly folliculocentric, inverse, and hyperkeratotic variants, highlights the considerable clinical diversity of PV and the potential for these unusual presentations to mimic other dermatoses, thereby complicating clinical diagnosis.

### 3.3. The Back Region (Upper Trunk) Was the Most Frequently Affected Anatomical Site in Patients with PV

The anatomical distribution of PV lesions demonstrated a distinct predilection for the upper trunk and other sebaceous-rich regions of the body (Supplementary Figure S1). Among the recorded anatomical sites, the back was the most frequently involved region, accounting for 23.8% (62) of lesion sites. This was followed by the neck, with 16.9% (44) sites, and the chest, with 14.9% (39) sites. Collectively, these three anatomical regions accounted for 55.6% (145) of the 261 affected sites, indicating that more than half of all lesions were concentrated over the upper trunk. In addition, the arms represented another commonly affected region, with 12.6% (33) of lesion sites, while the face accounted for 10.7% (28) of lesion sites. Together with the back, neck, and chest, these areas constituted the principal anatomical locations involved in PV, highlighting a marked preference for exposed and sebaceous areas of the body. Lesions were comparatively less frequent in other anatomical regions. The trunk (excluding the upper back and chest) accounted for 6.5% (17) of the affected sites, whereas the bilateral axilla contributed 5.0% (13) of the sites. In contrast, involvement of the abdomen was relatively uncommon, with only 3.4% (9) of affected sites. The legs and shoulders each accounted for 2.7% (7) of sites, while the ears represented the least frequently affected anatomical location, with only 0.8% (2) recorded sites.

When grouped according to body regions, lesions were predominantly distributed over the trunk, including the back, chest, neck, abdomen, shoulders, axillae, and trunk, which collectively represented the most affected sites. In contrast, involvement of the extremities (arms and legs) and craniofacial region (face and ears) was comparatively less common. This pattern demonstrates a clear concentration of lesions over the central body regions, particularly the upper trunk. The concentration of lesions within sebaceous-rich anatomical regions highlights the characteristic topographical distribution of PV among patients included in the study. Interestingly, the anatomical distribution of lesions varied with age. Among patients aged below 15 years (24), the face was the most involved anatomical site, occurring in 58.3% (14/24), followed by the back in 33.3% (8/24), consistent with previous findings [39]. When the other age groups were considered together, the back was consistently the predominant site of involvement, followed by the neck and chest. The frequency of infection in the back trunk area was particularly prominent in older age groups, indicating a clear shift from predominantly facial involvement in younger patients to greater involvement of the back, neck, and chest with increasing age.

### 3.4. M. furfur Was the Predominant Malassezia Species Isolated from Patients with PV

In our study, 147 patients diagnosed with PV yielded a total of 182 *Malassezia* isolates, which were classified into eight distinct *Malassezia* species (Figure 3). The species distribution showed a heterogeneous *Malassezia* population; however, *M. furfur* was clearly predominant. Of the 182 isolates recovered, 51.1% (93) were identified as *Malassezia furfur*, indicating that more than half of all characterized isolates belonged to this dataset. The second most frequently isolated species was *Malassezia japonica*, accounting for 34.1% (62) of isolates. Although its prevalence was considerably lower than that of *M. furfur*, *M. japonica* accounted for approximately one-third of all identified isolates and was substantially more prevalent than the other species. Collectively, *M. furfur* and *M. japonica* accounted for 85.2% (155) of the 182 isolates, indicating that these two species constituted the overwhelming majority of the *Malassezia* population associated with PV at the study location.

The remaining six species were isolated at markedly lower frequencies. *Malassezia restricta* was identified in 5.5% (10) of isolates, making it the third most prevalent species detected during the study. *Malassezia slooffiae* was recovered from 3.3% (6) of isolates, while *Malassezia sympodialis* and *Malassezia arunalokei* were each represented by 2.2% (4) of isolates. *Malassezia globosa* was comparatively uncommon, with only 1.1% (2) of isolates identified, whereas *Malassezia pachydermatis*, which is primarily zoophilic, was the rarest species encountered, represented by a single isolate (0.6%, 1 isolate) (Figure 3). The isolation of *M. pachydermatis* in this case may reflect the patient’s close contact with a pet dog, a plausible source of zoonotic transmission. The distribution of isolates revealed a pronounced decline in species frequency beyond the two dominant species. While *M. furfur* alone accounted for more than half of the total isolates, the cumulative contribution of the remaining six species was limited to 14.8% (27) of the isolates.

In addition to the identified *Malassezia* species, our molecular analysis revealed the coexistence of other opportunistic yeast species in a subset of samples. A total of 20 non-*Malassezia* yeast isolates were detected, comprising 15 *Candida parapsilosis* isolates, 2 *Rhodotorula mucilaginosa* isolates, and 1 each of *Candida orthopsilosis*, *Meyerozyma caribbica*, and *Trichosporon asahii* (Supplementary Table S3).

### 3.5. PV Was Most Prevalent Among Young and Middle-Aged Adults, with Age Influencing the Distribution of Malassezia Species

Age-stratified molecular analysis revealed PV mostly affected adolescents and young adults (10- to 29-year-old individuals), with distinct variations in the distribution of *Malassezia* species across different age groups (Supplementary Figure S2A,B). Although *Malassezia furfur* was isolated from patients of all ages, the diversity and abundance of the remaining species differed considerably between age categories. The 20–29-year-old age group exhibited the greatest diversity of *Malassezia* species, with all major species identified. In addition to the predominance of *M. furfur* and *M. japonica*, this age group also harbored *M. restricta*, *M. arunalokei*, *M. slooffiae*, and *M. sympodialis*, indicating the broadest species composition among all age categories. A similarly diverse distribution was observed in the 10–19-year-old age group, where *M. furfur* and *M. japonica* predominated, accompanied by occasional isolation of *M. restricta*, *M. globosa*, *M. slooffiae*, and *M. sympodialis*. Among patients aged 30–39 years, the species profile was comparatively less diverse, consisting predominantly of *M. furfur* and *M. japonica*, together with smaller numbers of *M. arunalokei*, *M. restricta*, and the only isolate of *M. pachydermatis* recovered during the study. In contrast, patients aged 40–49 and 50–59 years showed a marked reduction in species diversity, with infections being largely attributable to *M. furfur* and *M. japonica*, while only isolated occurrences of other species were observed. The pediatric and elderly age groups demonstrated the lowest species diversity. Patients aged 0–9 years yielded only *M. furfur* and *M. sympodialis*, whereas those aged 60–69 and 70–80 years were represented exclusively by *M. furfur*. None of the remaining *Malassezia* species were identified in these older age categories.

### 3.6. Male Patients Showed a Higher Prevalence of PV, While the Distribution of Malassezia Species Was Comparable Between Genders

Gender-wise analysis of the isolates demonstrated that although PV occurred more frequently among male patients, the overall composition of *Malassezia* species remained largely comparable between males and females (Supplementary Figure S3A). In both genders, *Malassezia furfur* was the predominant species, followed by *Malassezia japonica*, together accounting for most isolates. Among male patients, *M. furfur* accounted for the largest proportion of isolates, with *M. japonica* the second most frequently recovered species. The remaining species, including *M. restricta*, *M. slooffiae*, *M. arunalokei*, *M. sympodialis*, *M. globosa*, and *M. pachydermatis*, were identified at comparatively lower frequencies. Despite their lower prevalence, all eight *Malassezia* species identified in the study were represented among male patients, reflecting the greater number of isolates recovered from this group. A similar distribution pattern was observed among female patients. *M. furfur* remained the predominant species, followed by *M. japonica*, whereas the remaining species were recovered only occasionally. The relative abundance of individual species closely resembled that observed in males. Comparison between genders revealed no apparent gender-specific preference for any *Malassezia* species. Although the absolute number of isolates was higher in males due to the greater number of male patients, the proportional distribution of species remained comparable. In both genders, *M. furfur* accounted for approximately half of all isolates, followed by *M. japonica*, while the remaining species collectively represented only a small proportion (Supplementary Figure S3B).

### 3.7. Seasonal Variation Significantly Influenced the Epidemiology of Malassezia Species in PV

Seasonal analysis revealed marked temporal variations in both PV occurrence and *Malassezia* species distribution throughout the study period, with distinct patterns emerging between the two seasons examined. The April–September (summer) period accounted for the majority of clinically diagnosed PV cases and the greatest species diversity (90 cases). In contrast, the October–March (winter) period was characterized by an overall decline in case numbers, with case counts remaining comparatively lower (57 cases) (Supplementary Figure S4A).

*M. furfur* was recovered throughout most of the year and remained the predominant species in both periods, with peak isolation occurring across several months of the April–September period as well as the early part of the October–March period. *M. japonica* showed greater seasonal variation, occurring sporadically during the early months of the winter period, then increasing as the summer progressed, reaching its peak toward the end of that period, and declining thereafter into the following winter. *M. restricta* and *M. slooffiae* were recovered mainly at the boundary between the two periods, with recovery windows spanning the early months of the October–March period and the closing months of the April–September period, whereas *M. arunalokei*, *M. globosa*, *M. pachydermatis*, and *M. sympodialis* were isolated only occasionally, without a consistent seasonal pattern. Overall, the less prevalent species exhibited greater seasonal variability than *M. furfur* and *M. japonica*, which together sustained the predominant species burden throughout both periods (Supplementary Figure S4B).

Species richness also varied considerably between the two periods. Within the April–September period, the greatest diversity was observed toward its end, with almost all identified *Malassezia* species represented, corresponding to the peak PV case burden, whereas the earlier and middle months of this period showed comparatively reduced species diversity. Within the October–March period, the lowest species diversity of the entire study was recorded toward its latter half, with only a single species identified, reflecting the minimal disease burden during that time.

### 3.8. Potential Predisposing Factors Identified Among Patients with PV

A total of 18 patients with PV had documented underlying medical conditions or potential predisposing factors that may have contributed to the development or persistence of the disease (Supplementary Table S4). Alcohol dependence syndrome was the most frequently observed associated condition among the patients, whereas each of the remaining conditions was documented in individual patients. Alcohol-related disorders constituted a considerable proportion of the associated conditions. These included alcohol dependence syndrome, alcoholic liver disease with acute pancreatitis, liver cirrhosis with hepatic encephalopathy, and decompensated chronic liver disease associated with underlying coagulopathy, Grade I hepatic encephalopathy, alcoholic hepatitis, and macrocytic anemia. This indicates that chronic alcohol use and hepatic dysfunction were common systemic comorbidities among affected individuals. In addition, several patients had disorders associated with immune dysregulation or immunosuppression, including post-transplant lymphoproliferative disease, renal transplantation, chronic myeloid leukemia, discoid lupus erythematosus, and severe anemia. Respiratory illnesses were also recorded, including bronchiectasis with tuberculosis and bronchial asthma. Several patients presented with concomitant dermatological disorders, including tinea corporis with tinea cruris, tinea incognito, psoriasis vulgaris, lichen planus, and hand eczema (Supplementary Table S4). The patient with psoriasis vulgaris had been treated with a potent topical corticosteroid containing clobetasol, the patient with hand eczema had received topical betamethasone therapy, and the patient with lichen planus had a history of treatment with cyclosporine tablets. Additionally, the patient with chronic myeloid leukemia was receiving imatinib therapy. One patient also had a history of rickettsial fever. Significantly, there was a positive family history of PV among 13 affected patients, indicating that some had first- or second-degree relatives who had previously been affected by the condition. The finding suggested that familial clustering may be a risk factor for disease development, perhaps due to genetic predisposition or shared environmental conditions and hygiene practices. The above findings showed that PV affected individuals with various systemic diseases and dermatological disorders, as well as those receiving immunosuppressive therapy (Supplementary Table S4).

### 3.9. Phylogenetic Analysis Reveals Strain-Level Heterogeneity Among Clinical Malassezia Isolates

A maximum-likelihood phylogenetic tree based on ITS sequences was constructed using 33 clinical isolates (Supplementary Data S1) and eight *Malassezia* type-strain reference sequences to assess species-level relationships and strain diversity (Figure 4). Most isolates clustered with their corresponding type strains, forming well-supported species-specific clades. The *M. furfur* isolates formed a monophyletic group with moderate to strong bootstrap support, indicating overall genetic consistency within the species. Similarly, *M. sympodialis* and *M. slooffiae* isolates clustered into highly supported clades with limited intraspecific variation. The two *M. globosa* isolates grouped with the type strain, although one isolate exhibited greater sequence divergence. Notable heterogeneity was observed within the *M. furfur*–*M. japonica* complex. One isolate initially identified as *M. furfur* (CS 180-2) clustered within the *M. japonica* lineage, suggesting possible species-assignment discordance or close genetic relatedness between these taxa. Furthermore, *M. japonica* isolates formed two distinct sub-lineages, highlighting considerable genetic diversity within the species. A single isolate (CS 29) clustered strongly with the *M. pachydermatis* type strain. The *M. restricta* clade showed poor internal resolution, consistent with the low ITS sequence variability reported for this species. Nevertheless, *M. restricta* remained distinct from the closely related *M. arunalokei* clade, supporting their classification as separate species despite their close phylogenetic relationship. Overall, the phylogeny confirmed the species identity of most isolates while revealing notable strain-level heterogeneity and occasional species-assignment discordance within the *M. furfur*–*M. japonica* complex. The analysis also highlighted the close evolutionary relationship between *M. restricta* and *M. arunalokei*, while maintaining clear species-level separation.

## 4. Discussion

Skin diseases are known to adversely affect quality of life and are usually caused by microbial colonization and host immunity [40,41]. With the growing problem of antimicrobial resistance, accurate, species-level identification is crucial for improving treatment efficiency and reducing relapse rates [42,43]. *Malassezia* species have gained considerable interest because they are both commensals and pathogens and are suspected to be causative agents of various systemic diseases [37,38,44,45,46]. Given the differences in their pathogenicity, it is necessary to understand the distribution of these organisms across regions. Thus, the current study focused on species-level identification of *Malassezia* isolated from patients with PV in Dakshina Kannada, a poorly explored area of southern India. There is considerable geographic variability in the distribution of *Malassezia* species. *M. sympodialis* and *M. globosa* are prevalent in Europe [47,48,49], while in Asia, *M. restricta*, *M. globosa*, and *M. furfur* are most frequently isolated [50,51,52]. Several Indian studies have reported varying patterns of dominance by *Malassezia* species across study areas [23,24,25,26,27,28,29,30]. In contrast to numerous studies showing the prevalence of *M. globosa* or *M. sympodialis*, our study showed the predominance of *M. furfur* and *M. japonica*, which was confirmed by PCR-RFLP and Sanger sequencing (Supplementary Figure S5) (Supplementary Data S1). Likewise, similar dominance of *M. furfur* has been noted in various parts of tropical India [53,54,55,56,57,58,59], suggesting that climate may have a considerable effect on its distribution. These results underscore the need for region-specific epidemiological studies. The phylogenetic analysis revealed further heterogeneity at the strain level in certain species. The similarities found in *M. furfur*–*M. japonica* and *M. restricta*–*M. arunalokei* are in agreement with previously published findings [60,61]. The presence of intra-species diversity and occasional mismatches in species assignment reveals the complexity of species genomes and indicates the limitations of ITS sequencing in discriminating closely related strains. Conventional phenotypic methods based on morphological and physiological characteristics are simple and inexpensive but have limited discriminatory power because of overlapping phenotypic traits among closely related *Malassezia* species [62,63]. Molecular approaches have markedly improved species identification. ITS-based PCR assays are widely used for their high sensitivity and reproducibility, while PCR-RFLP offers a rapid and cost-effective method for differentiating common species [64]. However, both methods have limited resolution for phylogenetically related species with highly similar ITS sequences, and PCR-RFLP performance depends on the choice of restriction enzymes, which can lead to misclassification. Sequencing of the ITS and D1/D2 ribosomal DNA regions provides superior taxonomic resolution and remains the reference standard for confirming species identity and identifying uncommon or novel taxa [65]. Species-specific multiplex PCR enables rapid detection of multiple clinically relevant species but is restricted to those included in the primer panel and requires careful primer design to minimize cross-reactivity [66]. More recently, next-generation sequencing (NGS) and metagenomic approaches have enabled comprehensive characterization of the cutaneous mycobiome by detecting mixed fungal communities, unculturable organisms, and low-abundance species [67]. Despite their superior sensitivity and resolution, their routine diagnostic use remains limited by high costs, specialized instrumentation, and the need for bioinformatics expertise.

These properties may be considered responsible for the dominance of *M. furfur* in Dakshina Kannada. In tropical countries, the abundance of sebaceous glands and environmental moisture should favor the proliferation and survival of the organism [27,68,69]. Interestingly, *M. japonica* accounted for 34.1% of all isolates in our study group, revealing a high prevalence of this species [54,61,70,71,72,73,74,75,76,77,78] (Supplementary Table S5). This can be associated with climate conditions, urbanization [52], and the application of molecular identification methods, which can distinguish closely related species such as *M. furfur* and *M. japonica* [61,79]. Older reports, which mostly used phenotypic approaches for differentiation [16,50,80,81], may have underestimated the prevalence of *M. japonica.* The highest PV percentage was observed in young adults, which may be explained by increased sebaceous gland activity at this age [82,83]. Male predominance is due to differences in sebum production, lifestyle, occupation, and seeking medical help [84].

One of the interesting findings from this research is the presence of multiple fungi within PV lesions. In addition to *Malassezia* yeast, *Candida*, *Rhodotorula*, *Meyerozyma*, and *Trichophyton* species were identified (Supplementary Table S3). Moreover, several *Malassezia* species were found in the same lesion. PV can be considered a polymicrobial fungal ecosystem rather than a monospecific infection. The interactions between microorganisms can play an important role in disease pathogenesis and therapy response. PV-causing *Malassezia* proliferates more under favorable environmental and host conditions, particularly high temperature, increased humidity, and enhanced sebum production [85]. As a coastal city, Mangalore experiences a prolonged southwest monsoon characterized by persistently high relative humidity (often exceeding 80%) and warm tropical temperatures. The cumulative effects of these climatic conditions from August to October create an ideal microenvironment for *Malassezia* proliferation. Persistent moisture, increased perspiration, damp clothing, reduced evaporation, and limited sun exposure during the monsoon contribute to changes in the stratum corneum and alterations in skin pH and lipid composition, thereby facilitating fungal overgrowth [86]. Sustained exposure to these favorable environmental conditions, together with the inherently humid coastal climate, may explain the increased number of PV cases observed during September.

For localized PV, topical antifungal therapies such as selenium sulfide, zinc pyrithione, ketoconazole, and terbinafine are considered the first-line treatments [87]. In patients with extensive, recurrent, or refractory disease, systemic azole antifungals, particularly itraconazole and fluconazole, are the treatment of choice due to their efficacy against *Malassezia* species [71]. Adjunctive measures, including maintaining good skin hygiene, minimizing excessive sweating, and avoiding prolonged skin occlusion, are also recommended to reduce the risk of recurrence. The diseases that can be considered comorbid conditions in patients with PV included various alcohol-related problems, which were among the most common systemic diseases in our sample. Alcohol intake negatively affects the immune system and the state of the epidermis, increasing the likelihood of microbial colonization. Additionally, several patients had immunodeficiency conditions due to organ transplantation, hematologic malignancies, autoimmune diseases, or steroid treatment, all of which are related to impairment of immune regulation. There are several limitations associated with this study. First, being a single-center study, the results cannot be generalized to all populations. There is also potential bias due to culture-based isolation and a small sample size, which may have led to the failure to identify rare species. However, the results provide important information on the molecular epidemiology of *Malassezia* in southern India. Multicenter studies utilizing whole-genome sequencing, multilocus sequence typing, and metagenomics can help shed more light on the species diversity, population structure, mechanisms of pathogenesis, and relevance of *Malassezia* infections. A limitation of the present study is that antifungal susceptibility testing was not performed. Although the present study focused on the molecular epidemiology of *Malassezia*, future investigations should evaluate the antifungal susceptibility profiles of these clinical isolates to determine species-specific resistance patterns and their potential implications for therapeutic management and disease recurrence. Future studies with standardized antifungal susceptibility testing will be valuable for correlating species distribution with antifungal response and informing evidence-based treatment strategies.

## 5. Conclusions

In this study, the molecular epidemiology of *Malassezia*, a human commensal fungus that causes PV, in the coastal area of Karnataka, India, has been presented. Molecular identification of the isolates confirmed the predominance of *M. furfur*, along with a high prevalence of *M. japonica*, suggesting that the role of this species in the pathogenesis of PV was likely underestimated in previous studies, a bias primarily attributable to classical identification techniques and a similar ITS amplicon size. Furthermore, six other *Malassezia* species have been identified, illustrating the significant species diversity associated with PV. Epidemiologically, this condition is predominantly prevalent among adolescents and young adults and is more frequent in males. Despite the predominance of *M. furfur* across all age groups, genders, and seasons, differences in the distribution of the remaining *Malassezia* species confirmed the influence of demographic and seasonal factors on their epidemiology. In terms of clinical presentation, hypopigmented macules were the most frequent, while lesions mostly occurred on the back and other areas of the upper trunk with abundant sebaceous glands. Some of the patients were suffering from a disease or taking immunosuppressants or corticosteroids, indicating that host-related factors could contribute to predisposition to the disease. All these data confirm the usefulness of molecular tools for the accurate identification of *Malassezia* species and provide current information on the region’s epidemiology and can serve as a starting point for further multicenter studies on its geographical distribution and pathogenicity.

## Figures and Tables

**Figure 1:**
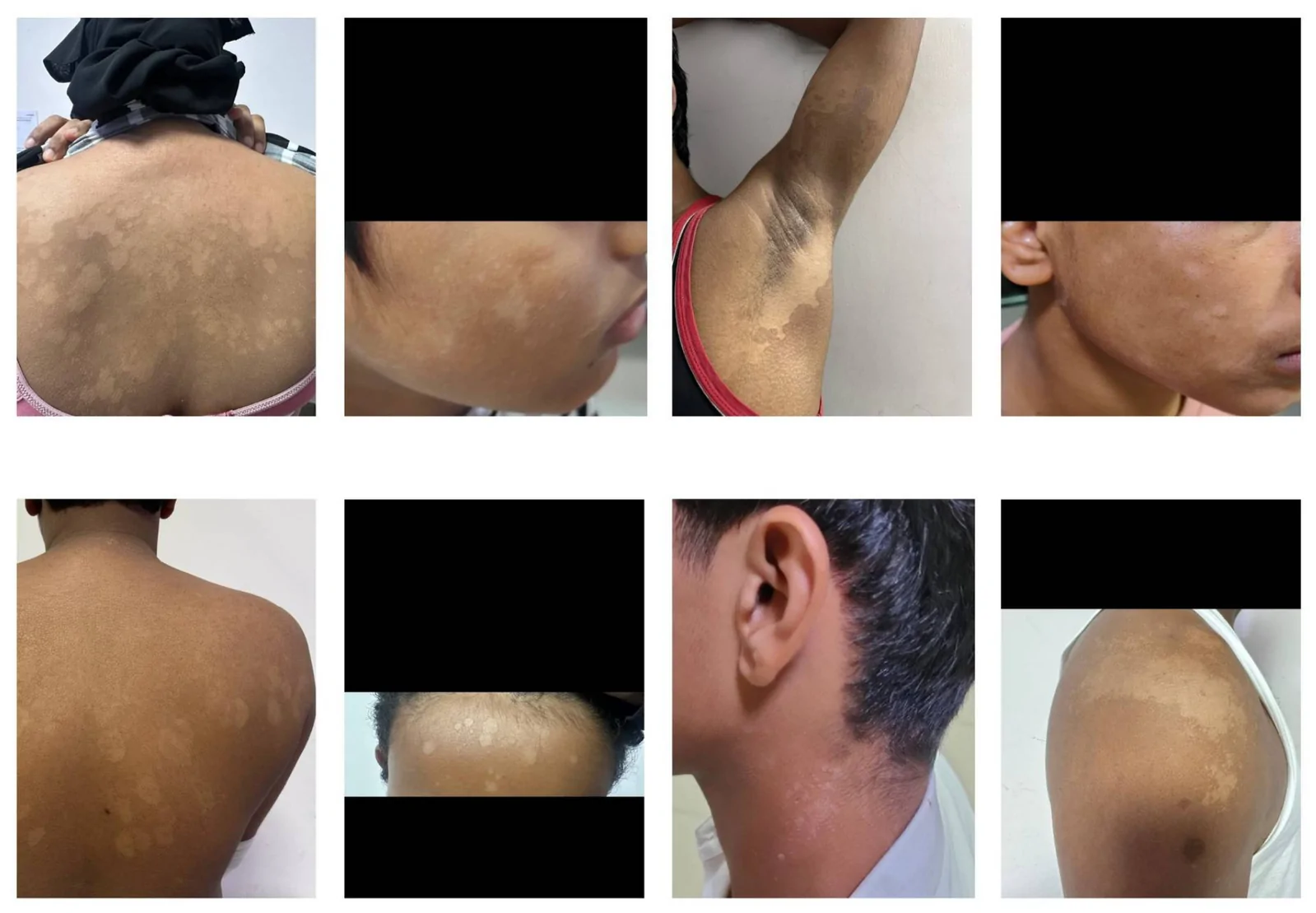
**Representative clinical presentations of patients enrolled in the study for *Malassezia*-associated infections.** Clinical images showing the diverse characteristics of PV in a study population, including hypopigmented and hyperpigmented lesions involving different anatomical sites such as the back, face, neck, axilla, forehead, and shoulder.

**Figure 2:**
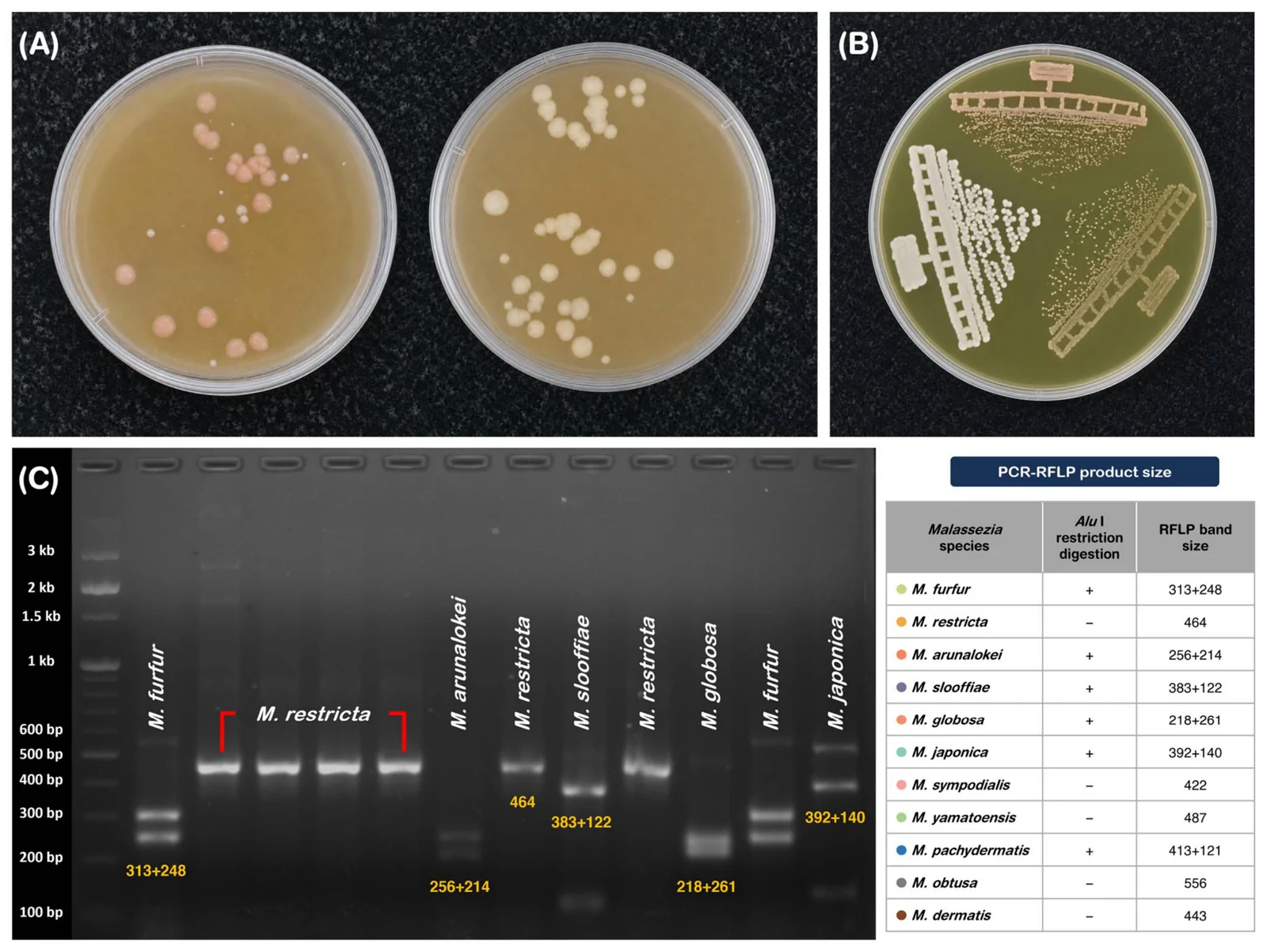
**Isolation, purification, and PCR–RFLP-based identification of *Malassezia* species.** Representative images of (**A**) Primary *Malassezia* colonies isolated from clinical specimens, (**B**) purified colonies obtained following subculturing, and (**C**) agarose gel electrophoresis showing species-specific *Alu* I-digested PCR-RFLP banding patterns of reference *Malassezia* strains.

**Figure 3:**
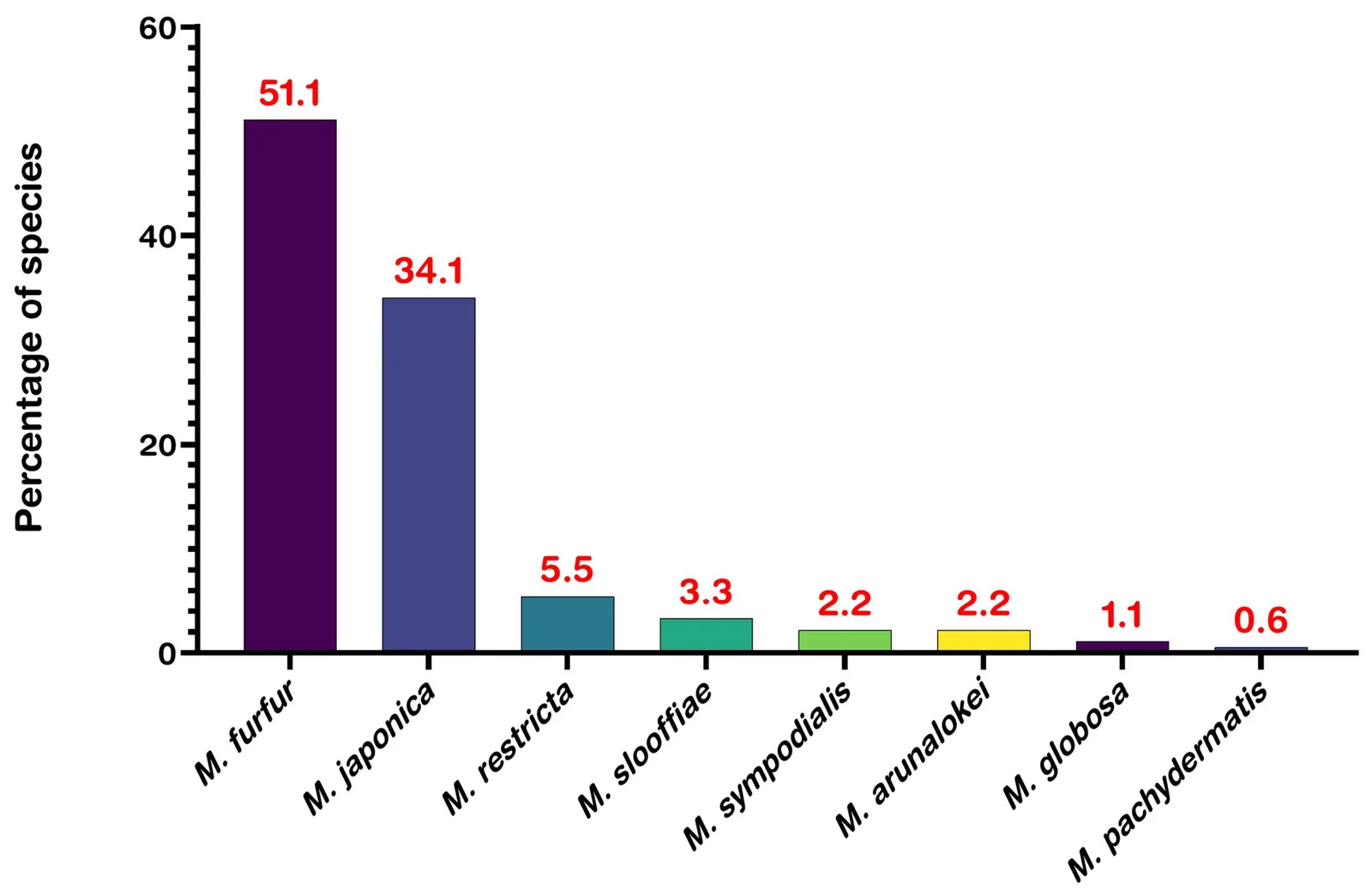
**Distribution of *Malassezia* species isolated from patients with PV.** Percentage distribution of *Malassezia* species identified from patients with PV by molecular characterization in our study population.

**Figure 4:**
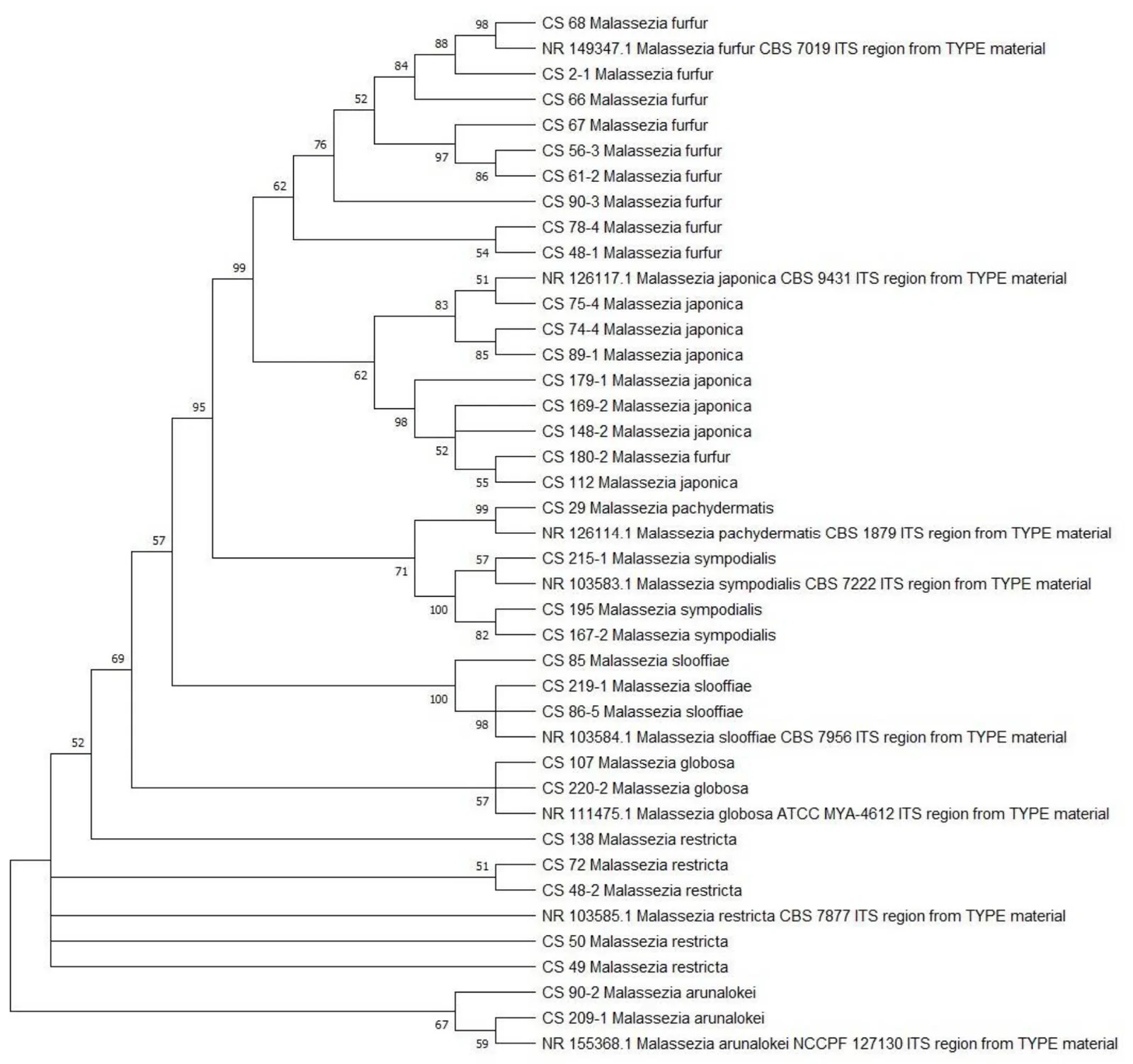
**Maximum-likelihood phylogenetic analysis of representative clinical *Malassezia* isolates based on ITS rDNA sequences**. Phylogenetic tree constructed using ITS rDNA sequences of representative *Malassezia* isolates recovered in the present study and corresponding reference/type strains retrieved from GenBank. The isolates clustered into distinct species-specific clades comprising *M. furfur*, *M. japonica*, *M. slooffiae*, *M. pachydermatis*, *M. sympodialis*, *M. globosa*, *M. restricta*, and *M. arunalokei*. Bootstrap values (≥50%) from 100 replicates are shown at the branch nodes, indicating the robustness of the inferred phylogenetic relationships.

## Data Availability

The data presented in this study are available on request from the corresponding author.

## Supplementary Materials

The following supporting information can be downloaded at https://www.mdpi.com/article/10.3390/microorganisms14102318/s1, Figure S1: Anatomical distribution of PV lesions among the study population; Figure S2: Age-wise distribution of PV cases and associated *Malassezia* species; Figure S3: Gender-wise distribution of patients with PV and the corresponding distribution of molecularly identified *Malassezia* species; Figure S4: Seasonal distribution of PV cases and the corresponding distribution of identified *Malassezia* species; Figure S5: Representative PCR-RFLP gel images of the clinical isolates; Table S1: Demographic characteristics of the study population with PV; Table S2: Distribution of clinical presentations among patients with PV; Table S3: Non-*Malassezia* species isolated from the PV lesions; Table S4: Underlying medical conditions and potential predisposing factors identified among PV patients; Table S5: Prevalence of *Malassezia japonica* reported in previous studies; Data S1: ITS region DNA sequence dataset of clinical isolates included in this study.
