# Supplementary material for: Molecular Epidemiology of Pityriasis Versicolor Reveals a Remarkably High Prevalence of Malassezia japonica in Coastal South India
Source: Microorganisms. 2026 Oct 12;14(10):2318. doi: 10.3390/microorganisms14102318 (PMC13707797; doi:10.3390/microorganisms14102318)
Supplement: Supplementary file 1 [file microorganisms-14-02318-s001.zip › microorganisms-4480212-supplementary.pdf]

Supplementary tables:

Supplementary Table S1: Demographic characteristics of the study population with PV

Characteristic	Category	Number of patients (n)	Percentage (%)
**Gender**	Male	101	68.7%
	Female	46	31.3%
**Age**	0-9	7	4.8%
	10-19	42	28.6%
	20-29	44	29.9%
	30-39	26	17.7%
	40-49	13	8.8%
	50-59	11	7.5%
	60-69	1	0.7%
	70-80	3	2.0%
**Mean Age (years)**	Mean ± SD	27.8 ± 14.9 years	-

Supplementary Table S2: Distribution of clinical presentations among patients with PV

Clinical presentation	Number of patients (n)	Percentage (%)
Hypopigmented macules	77	47.8%
Hyperpigmented macules	35	21.7%
Erythematous plaques	10	6.2%
Hyperpigmented plaques	7	4.3%
Hypopigmented patches	6	3.7%
Erythematous macules	6	3.7%
Erythematous papules	5	3.1%
Hypopigmented plaques	4	2.5%
Hyperpigmented papules	4	2.5%
Hypopigmented papules	3	1.9%
Hypopigmented lesions	3	1.9%
Hyperpigmented lesions	1	0.6%

Supplementary Table S3: Non-*Malassezia* species isolated from the PV lesions

Species	No of isolates
Candida parapsilosis	15
Rhodotorula mucilaginosa	2
Candida orthopsilosis	1
Meyerozyma caribbica	1
Trichosporon asahi	1

Supplementary Table S4: Underlying medical conditions and potential predisposing factors identified among PV patients

Underlying medical condition / Predisposing factor	Number of patients (n)
Alcohol Dependence Syndrome (ADS)	2
Alcoholic Liver Disease and Acute Pancreatitis	1
Bronchial asthma	1
Bronchiectasis and Tuberculosis	1
Chronic Myloid Leukemia	1
Decompensated Chronic Liver Disease (DCLD), Underlying coagulopathy, Grade I Hepatic Encephalopathy, Alcoholic hepatitis and Macrocytic anemia	1
Discoid Lupus Erythematosus	1
Hand Eczema	1
Lichen Planus	1
Liver Cirrhosis, Hepatic encephalopathy	1
Post-transplant lymphoproliferative disease (PTLD)	1
Psoriasis vulgaris	1
Renal Transplant	1

Rickettsial fever	1
Severe anaemia	1
Tinea corporis with Tinea cruris	1
Tinea Incognito	1

Supplementary Table S5: Prevalence of *Malassezia japonica* reported in previous studies

**S.No**	**Study location**	**Disease condition**	**Method of detection**	**Sample size**	***M. globosa***	***M. furfur***	***M. restricta***	***M. japonica***
1	Assam, India	PV	PCR-RFLP and 26 rDNA sequencing	290	9.4%	83.1%	2.8%	**0.34%**
2	Chandigarh, India	Psoriasis	PCR-RFLP	50	8.8%	70.6%		**11.8%**
3	Mysuru, India	Dandruff	Morphological and biochemical	300	21%	36%	18%	**8%**
4	Brazil	PV and SD	D1/D2 rDNA sequencing	102	15%	40%		**7.5%**
5	Brazil	PV	D1/D2 rDNA sequencing	16		30%		**30%**
6	China	SD	ITS-Sequencing	65	1.56%	76.56%		**9.38%**
7	Vietnam	PV	PCR-RFLP and D1/D2 rDNA sequencing	305		96.25%		**3.75%**
8	Indonesia	SD	Biochemical assays	61	52%		8.3%	**6.3%**
9	Turkey	Cephalic pustules	Biochemical assays	21		73.3%		**26.7%**
10	Vietnam	PV	Biochemical assays	300	42.4%	14.4%	4%	**4.1%**
11	Iran	SD	Biochemical assays	100	55.8%	32.5%	9.1%	**1.3%**
12	Japan	AD	Nested PCR	36				**33.3%**

M. sympodialis	***M. obtusa***	***M. slooffiae***	***M. dermatis***	***M. yamatoensis***	***M. pachydermatis***	***Others***	**References**
1.9%	2.46%						[73]
	2.9%	5.9%					[54]
12%	5%						[72]
27.5%		5%		5%			[77]
20%	10%	10%					[71]
12.5%							[78]
							[52]
4.2%	2.1%		22.9%			4.2%	[76]
							[70]
4.8%	5.9%	1.8%	17.3%		0.4%	4.9%	[75]
1.3%							[74]
						66.7%	[61]

Supplementary figures:

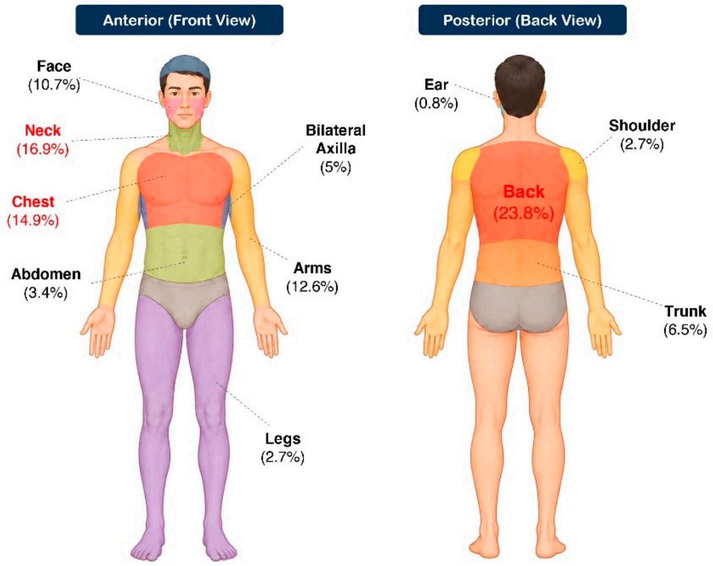

Summary of Body Site Involvement

Body site	No. of sites	Percentage (%)
Abdomen	9	3.4%
Arms	33	12.6%
Back	62	23.8%
Bilateral Axilla	13	5.0%
Chest	39	14.9%
Ear	2	0.8%
Face	28	10.7%
Legs	7	2.7%
Neck	44	16.9%
Shoulder	7	2.7%
Trunk	17	6.5%

Supplementary Figure S1: Anatomical distribution of PV lesions among the study population. Distribution of PV lesions across different anatomical sites maps depict lesion localization on the anterior and posterior aspects of the body, while the table summarizes the frequency and percentage of involvement at each anatomical site.

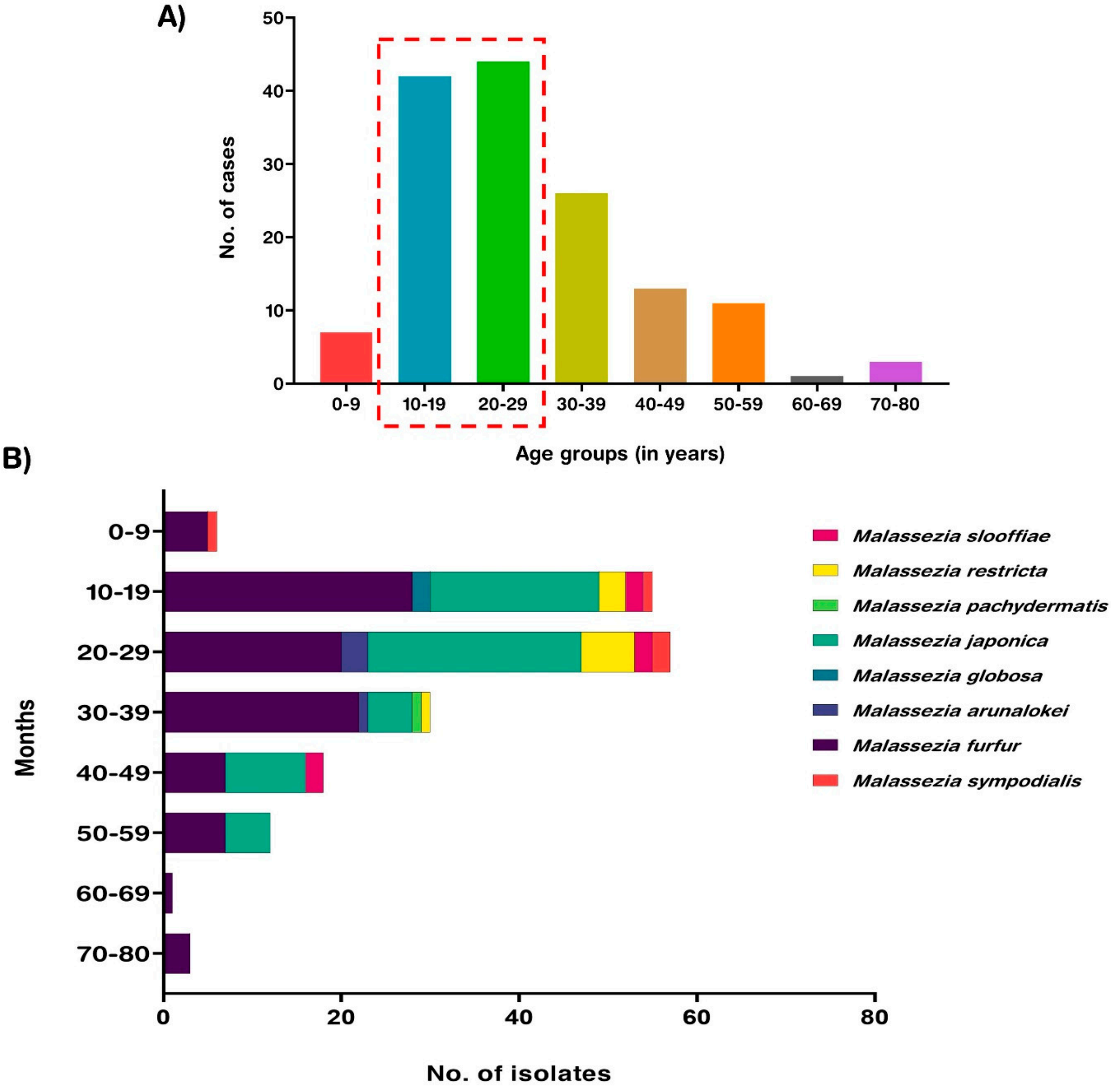

Supplementary Figure S2: Age-wise distribution of PV cases and associated *Malassezia* species. Distribution of patients with pityriasis versicolor according to age group. (B) Age-specific distribution of molecularly identified *Malassezia* species.

A)

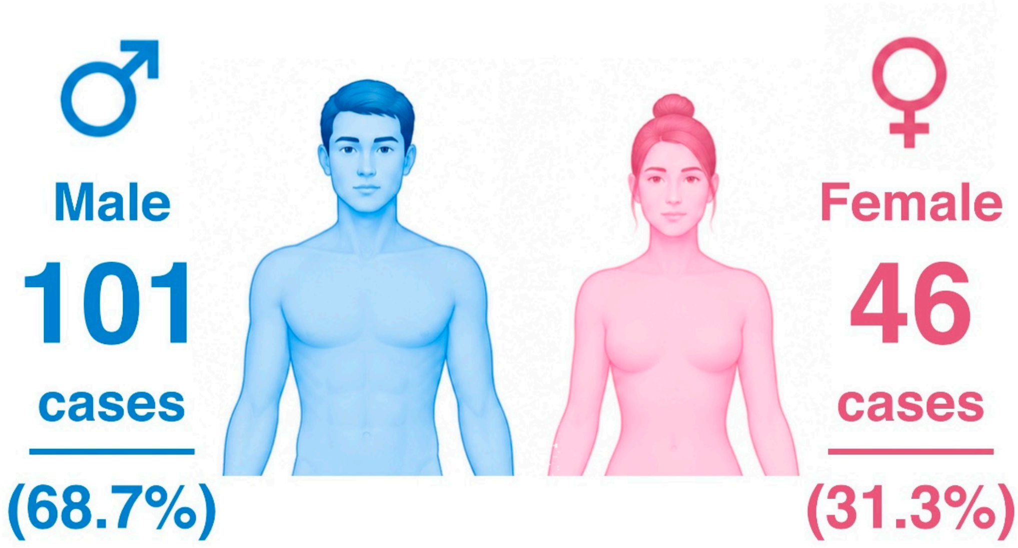

B)

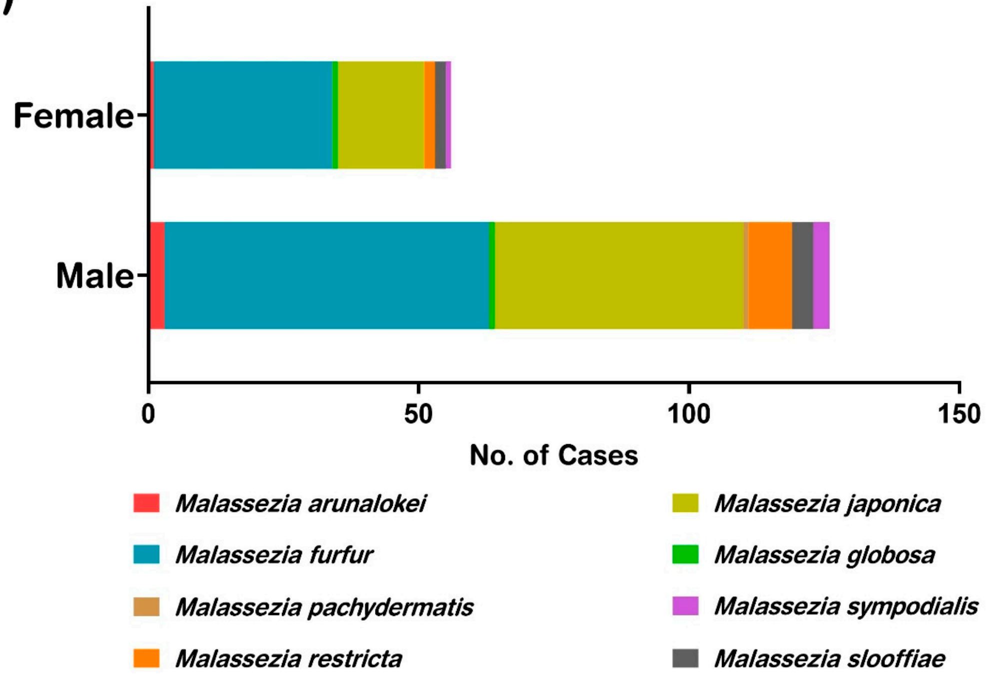

Supplementary Figure S3: Gender-wise distribution of patients with pityriasis versicolor (PV) and the corresponding distribution of molecularly identified *Malassezia* species. (A) Gender distribution of the study population, showing the number and percentage of male and female patients diagnosed with PV. (B) Distribution of the eight *Malassezia* species identified among male and female patients.

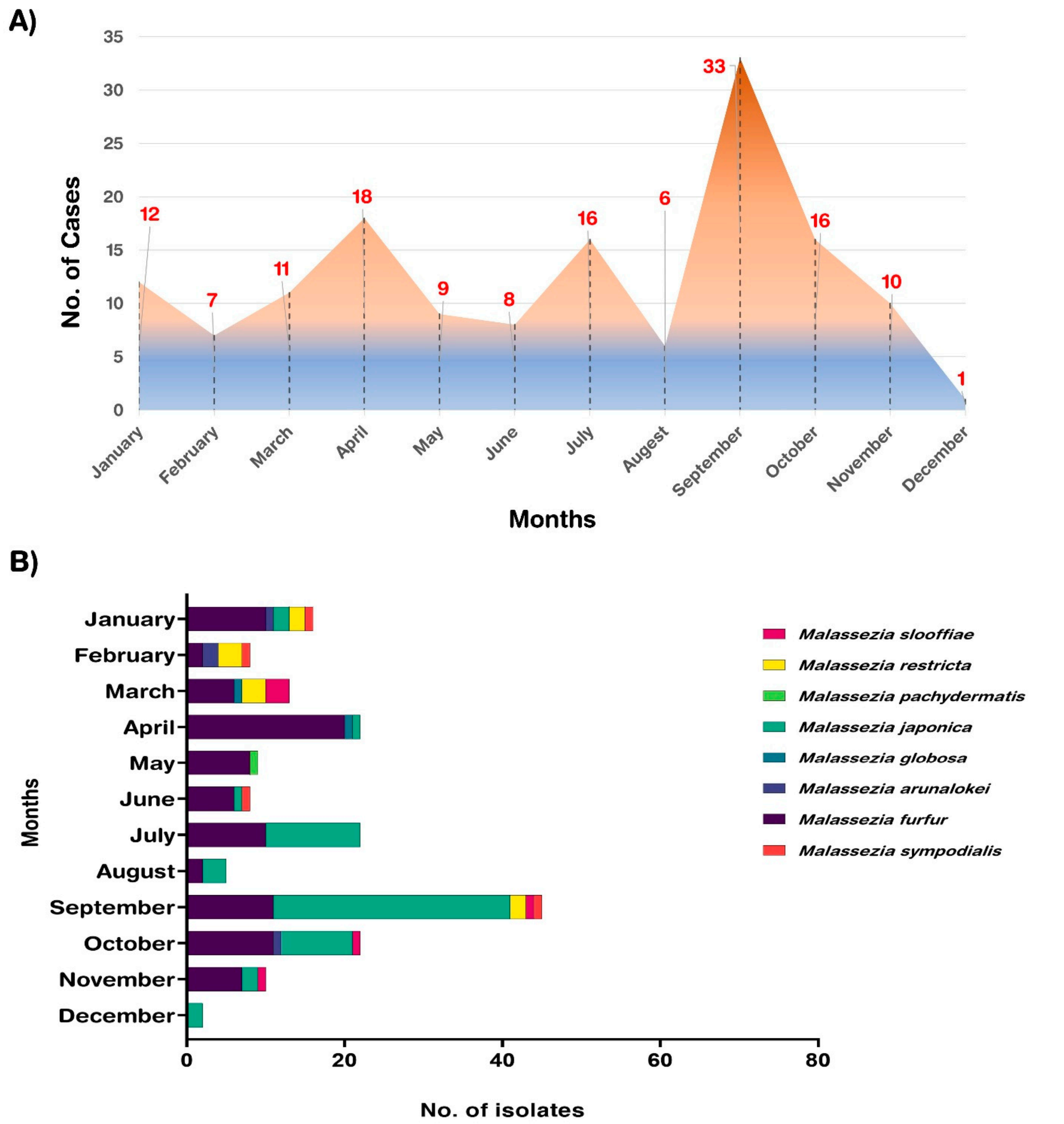

Supplementary Figure S4: Seasonal distribution of PV cases and the corresponding distribution of identified *Malassezia* species. (A) Number of clinically diagnosed PV cases recorded each month. (B) Monthly distribution of the *Malassezia* species identified from clinical isolates.

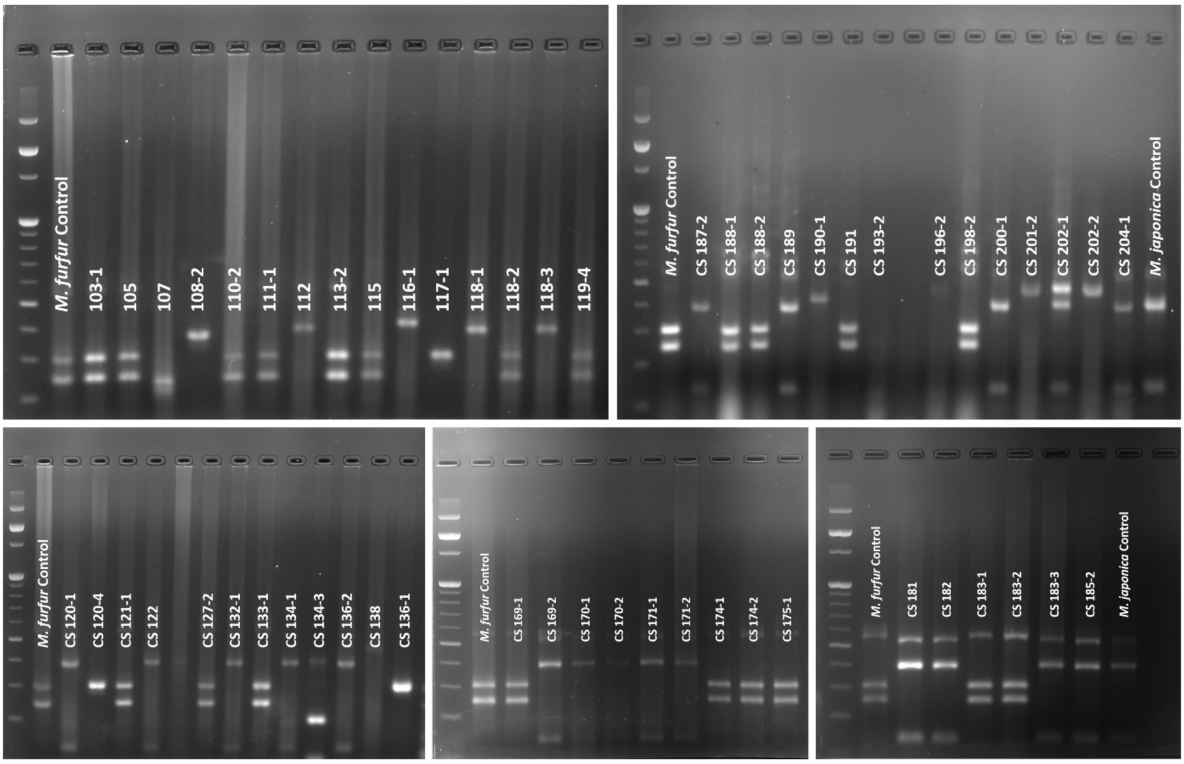

Supplementary Figure S5: Representative PCR-RFLP gel images of the clinical isolates. PCR-RFLP banding patterns obtained from clinical isolates following digestion of PCR-amplified ITS regions with the restriction enzyme *Alu I*.

Supplementary Data S1: ITS region DNA sequence dataset of clinical isolates included in this study.

>CS_29_Malassezia_pachydermatis

GGGGTGTGCGCTAGCCATACGTATCGCGCGAGCGTCATCTGTGCGCCCGAACCCACTATACATCCACAAACCCGTGTGCA

CTTGTGTTGCTTTGGCCTGTATGGGCGAGCGCACGCATTCAAACAAACTCGTATGGTTGTATGTACGTTGTAAACGTTGG

ACCGTCACTGGCCAACAAACTTTATACAACTTTCGACAACGGATCTCTTGGTTCTCCCATCGATGAAGAACGCAGCGAAA

CGCGATAGGTAATGTGAATTGCAGAATTCCGTGAATCATCGAATCTTTGAACGCACCTTGCGCTCCATGGTATTCCGTGG

AGCATGCCTGTTTGAGTGCCGCGAATTCTCCCACCCCAAACGGTTGCCGAAAGGTACTGTGCGGCGGAGGGGTTGGATGG

GTGCTACTGCCTGTGGTGGGGAAACTACAACAGGCTCGCCCGAAATGCATTAGCGCCTCAGGACACATTCGCTACTGCTC

TACAAAAGGAAGAGCAGCGAAGCGTATGGGGGGGAAAACCACCACCGGTTATTCCAAACCTCGCCTGACTGTTTTTGCGC

GCATGGCATGATACGTCATTTGCTGTGTTGCGTAGGTTGGTGCGTGGGACTGTGTGAGTGCCTCTAGAGCTTTGAGAAGT

GTGTTTCGTGTGCTACGAACTGAAAAAGTACATTCCTTTTTTTCAATTCTGGTCTCAAATCAGGTAGGATCACCCGCTGA

ACTTAAGCATATCATAGACCCGGAAGAAAAGACCGTTAGTGAATTTGTAGGACCTGCCAAAGGGATGCACAGACTCTGTG

CCCACCCACTATACATCCACAAACCCGTGTGCACTTGTGTTGCTTTTGCCCTATATGGCACACCCCCCTTCAAAAAAACT

C>CS_48-1_Malassezia_furfur

TGTCAGCGGTAAGTAACGTCAGGAGGTGCTGCTGCGGAGCATATCAATAAGCGGAGGAGGACCGTAGGAGAACCTGCGGA

GGATCTTATTAATCAGAGGAAGACGTTTGGACCTCGCCGAGGCCTGGCTCTCCAATCCATTTCTACCAACTCATATGGTT

GTATGAACGTGGAAATCGTTGGACCGTAACTGGCCAACAACCAATAATACAACTTTCGACAACGGATCTCTTGGTTCTCC

CATCGATGAAGAACGCAGCGAAACGCGATAGGTAATGTGAATTGCAGAATTCCGTGAATCATCGAATCTTTGAACGCACC

TTGCGCTCCATGGTATTCCGTGGAGCATGCCTGTTTGAGTGCCGTGAATTCTCTCTCCCCAAGCGGTTGCGATTGCACTG

CTTTGGCGGACGAGGTTGGATGGGTGCTTCTGCCTGTTTCACAAGAAACAGGCTCGCCCGAAATGCATTAGCGCCTTTGG

GACACACTCTGCAAACCGCTCTGAAAGGGAGGGCGGCAGAGGGGATGGAGGAACTCCGCCCGTCAGCTATACCAAACTTT

GCCCCAGGCAAGCGCATGGCATGATACGTCATTTGCTGTGTGTGCGTCCTGAGGCAAGGGGCCGATGTGGTGCCTTTGTC

ACTCTGTGGGTGTGGTTGGTGCGCTACCAATCCTGCGTGGGAGGCTGGTCTGGTGCGCCAGCATGGAAGCAGGTGCGTGG

AAACGCGATTCCCTTTTATTCGTGTCGGGCGCGCGAGTCAGGGGGGATGGACCCGGCTGAAAGTTAACGTCTCTTTAGCG

GAGGAGAGAGGATCTTAGGGAAACCTACGCTATCCGTTCTGAACGGCCTGTTCCAGCAGG

>CS_48-2_Malassezia_restricta

CTTAGGCGGGAAAGTACGTCCGGTGGTCCAGCTGCGGAAGTTTCTGTTTGCCGACGACGTTATTATCCGAAGCCCTACTG

CACTGTTTTGTCTAACGGCTTCTGAATACCTTTTTGCGGCCTCTCTTGGGGTCTTTCTTCTCAACAAACTCTAATGGTAT

CCACCAAACTGGAACTTGGCCGGACCTTCACTGGCCAACAAACTATACACAACTTTCAACCTTATATCTCTTGGATCTCC

CATCGATGAAGAACACAGCGAAACCTAATAAGTAATGATAATTATTTAATTCCCCGAATCATCTAATCTTTGAACGCACC

TTGCGCTCTATGGCAATCCCCTGAGCATGCCTGTTTGAGTGCCATGAAATCTCCCCCCCCCCCGGATTTTAAATGAAACC

GCTTGGCGGATGGGGCCTGAATGGGTGCCTCTGCCTGAGCTACCTAGCACAGGCTCACCATATATGCATCATCGCCTTCA

TACACTTGACAATCGCCTCTCTGTTTGGGAGGATGCGGCCAAGCTTTGAATCTTTCTCCTGCCATGGCCTCATACGTCAT

TTGCTAAGTCGCCTAAAGGAGGAATGTTTGGTTGTGTCTGCGTGTGCTTCGAACTTGCCTCTGTGGCACATCCCAATTTC

ACTTCTGGTCTCAAATCAGGTAGGATCACCCGCTGAAACTTAAGCATATCAATAAGCGGAGGAAAAGATCGTTGAGAAAA

TAGGGGCAGTCATTAGGACGCAAAAAGTGTGCGGGGCGCAACCTACTATAATAAAAACCATGATGCATATGTGAACAAAG

GGATCACAA

>CS_49_Malassezia_restricta

GGTAAAAATCGGGCATGTAGTACGTGGGTGCACGACGCCGAAAGTGTCCCTGGCCGCCTACACCCACTATACATCCACAA

ACCCGTGTGCACTGTCTTGGAGAAAGGCTTCAGAGAAGTTTTTTGTGGCCTCTCTTGGGGTCTTTCTTCGCTACAAACTC

GAATGGTTAGTATGAACGTGGAACTTGGTTGGACCGTCACTGGCCAACAAACTATACACAACTTTCGACAACGGATCTCT

TGGTTCTCCCATCGATGAAGAACGCAGCGAAACGCGATAGGTAATGTGAATTGCAGAATTCCGTGAATCATCGAATCTTT

GAACGCACCTTGCGCTCTATGGCAATCCGTAGAGCATGCCTGTTTGAGTGCCATGAAATCTCCCACCCCAAGCGGTTTTT

AAATGAAACGGCTTGGCGGATGGGGTCTGGATGGGTGCCTCTGCCTGCGCTACCTAGCACAGGCTCGCCCGAAATGCATG

AGCGCCTTGAGACACTTTGCATCCGCCTCTCTGTTTGGGAGGAGGCGGCCAAGCAGTGTTTTTCTCCTGGCATGGCATGA

TACGTCATTTGCTATGTCGCCTAAAGGAGGAATGTTTGGTTGTGTCTGCGTGTGCTTCGAACTTGCCTCTGTGGCACATC

CCAATTTCACTTCTGGTCTCAAATCAGGTAGGATCACCCGCTGAACTTAAGCATATCAATAAGCGGAGGAA

>CS_50_Malassezia_restricta

TGGTCAAGGAGGTACGTCCGGAGGACCTGCTGCAGGAGCTATATCAATAAGCGGAGGAGCGTCCGGAGGTGATGCTGCGG

AGGACATTGTTAATCGGAGGAGGTTCAGAGAAGTTTTTTGTGGCCTCTCTTGGGGTCTTTCTTCGCTACAAACTCGAATG

GTTACTATGAACGTGGAACTTGGTTGGACCGTCACTGGCCAACAAACTATACACAACTTTCGACAACGGATCTCTTGGTT

CTCCCATCGATGAAAAACGCAGCGAAACGCGATAGGTAATGTGAATTGCAGAATTCCGTGAATCATCGAATCTTTGAACG

CACCTTGCGCTCTATGGCAATCCGTAGAGCATGCCTGTTTGAGTGCCATGAAATCTCCCCCCCCAAGCGGTTTTTAAATG

AAACGGCTTGGCGGATGGGGTCTGGATGGGTGCCTCTGCCTGCGCTACCTATCACAGGCTCGCCCGAAATGCATGAGCGC

CTTGAGACACTTTGCATCCGCCTCTCTGTTTGGGATGAGGCGGCCAATCAGTGTTTTTCTCCTGGCATGGCATGATACGT

CATTTGCTATGTCGCCTAAAAGGAGGAATGTTTGGTTGTGTCTGCGTGTGCTTCAAACTTGCCTCTGTGGCACATCCCAA

TTTCACTTCTGGTCTCAAATCAGGTAGGATCACCCGCTGAACTTAAGCATATCAATAAGCGGGAGGGAGAGGAATGTTAG

TTGAGAATTCGGCAAGGCCATACGAACGCAAAGACGTGGTCCTGGACCTACCCGCTATACATCACGACCCGGTGGAGCAG

TGGCTGGAGAAAAGCGTTTCGAAGAGATCTCTA

>CS_56-3_Malassezia_furfur

TCAAGTAGGCAGAGTCGTAGGAGGAGCTGCGGCAGCTCTCATAATCGGCGGAGGAGCCCAACTTTACACAATATCCACAA

ACCCGTGTGCACCGTTTGGATGAGTTGGACCTCGCAAGAGGCTCGGCTCTCCAATCCATTTCTACCAAACTCGTATGGCT

GTATGAACGTGGAAATCGTTGGACCGTAACTGGCCAACAACCAATAATACAACTTTCGACAACGGATCTCTTGGTTCTCC

CATCGATGAAGAACGCAGCGAAACGCGATAGGTAATGTGAATTGCAGAATTCCGTGAATCATCGAATCTTTGAACGCACC

TTGCGCTCCATGGTATTCCGTGGAGCATGCCTGTTTGAGTGCCGTGAATTCTCTCTCCCCAAGCGGTTGCGATTGCACTG

CTTTGGCGGACGAGGTTGGATGGGTGCTTCTGCCTGTTTCGCAAGAAACAGGCTCGCCCGAAATGCATTAGCGCCTTTGG

GACACACTCTGCAAACCGCTCTGAAAGGGAGGGCGGCAGAGGGGATGGAGGAACTCCGCCCGTCAGCTATACCAAACTTT

GCCCCAGGCAAGCGCATGGCATGATACGTCATTTGCTGTGTGTGCGTCCTGAGGCAAGGGGCCGATGTGGTGCCTTTGTC

ACTCTGTGGGTGTGTTGGTGCGCTACCAATCCTGCGTGGAGGCTGGTCTGTGCGCAAGCATGGAGCAGTTCTTGTGAACG

CATTCCCTTTTTCATTTCTGGTCTCAAATCAGGTAGGATCACCCGCTGAACTTAAGCATATCAATAAGCGGAGGAAAGGA

TCA

>CS_61-2_Malassezia_furfur

GGCTTAGTAGGCAGAGTACGTAGGAGAGCTGCGGCAGCTCTCATAAACGGCGGAGGAGCCCAACTTTACACAATATCCAC

AAACCCGTGTGCACCGTTTGGATGAGTTGGACCTCGCAAGAGGCCTGGCTCTCCAATCCATTTCTACCAAACTCGTATGG

TTGTATGAACGTGGAAATCGTTGGACCGTAACTGGCCAACAACCAATAATACAACTTTCGACAACGGATCTCTTGGTTCT

CCCATCGATGAAGAACGCAGCGAAACGCGATAGGTAATGTGAATTGCAGAATTCCGTGAATCATCGAATCTTTGAACGCA

CCTTGCGCTCCATGGTATTCCGTGGAGCATGCCTGTTTGAGTGCCGTGAATTCTCTCTCCCCAAGCGGTTGCGATTGCAC

TGCTTTGGCGGACGAGGTTGGATGGGTGCTTCTGCCTGTTTCGCAAGAAACAGGCTCGCCCGAAATGCATTAGCGCCTTT

GGGACACACTCTGCAAACCGCTCTGAAAGGGAGGGCGGCAGAGGGGATGGAGGAACTCCGCCCGTCAGCTATACCAAACT

TTGCCCCAGGCAAGCGCATGGCATGATACGTCATTTGCTGTGTGTGCGTCCTGAGGCAAGGGGCCGATGTGGTGCCTTTG

TCACTCTGTGGGTGTGTTGGTGCGCTACCAATCCTGCGTGGAGGCTGGTCTGTGCGCAAGCATGGAGCAGTTCTTGTGAA

CGCATTCCCTTTTTTCATTTCTGGTCTCAAATCAGGTAGGATCACCCGCTGAACTTAAGCATATCAATAACGCGGAGGAA

AGGATCATTAGTGAAAGCAAGGGCCAGCCATACGGAAGGCGCTCTCGGTACACGTCTCTGGCGCCAACTTTAACAATATC

ACAAACCCGTGTGCACGTTTGGAATAAATTGGAACTCCAGAAGGCGGGCTCCATCTTTTCTCCAACCCTAGGGGTGGTAT

GAACGGGGAAAATCGTTGGGACGTTAACTGGGCCACCAAT

>CS_66_Malassezia_furfur

TTTTAGTTGGGCAGAGGTCGTAGGAGGGCTGGGGCAGCTCTCGTACAACGTGCAGGTAGGCGCCCAACTTTACACAATAT

CCACAAACCCGTGTGCACCGTTTGGATGAGTTGGGCAAGAGGCTCGGCTCTCCAATCCATTTCTACCAAACTCGTATGGT

TGTATGAACGTGGAAATCGTTGGATCGTAACTGGCCAACAACCAATAATACAACTTTCGACAACGGATCTCTTGGTTCTC

CCATCGATGAAGAACGCAGCGAAACGCGATAGGTAATGTGAATTGCAGAATTCCGTGAATCATCGAATCTTTGAACGCAC

CTTGCGCTCCATGGTATTCCGTGGAGCATGCCTGTTTGAGTGCCGTGAATTCTCTCTCCCCAAGCGGTTGCGATTGCACT

GCTTTGGCGGACGAGGTTGGATGGGTGCTTCTGCCTGTTTCGCAAGAAACAGGCTCGCCCGAAATGCATTAGCGCCTTTG

GGACACACTCTGCAAACCGCTCTGAAAGGGAGGGCGGCAGAGGGGATGGAGGAACTCCGCCCGTCAGCTATACCAAACTT

TGCCCCAGGCAAGCGCATGGCATGATACGTCATTTGCTGTGTGTGCGTCCTGAGGCAAGGGGCCGATGTGGTGCCTTTGT

CACTCTGTGGGTGTGTTGGTGCGCTACCAATCCTGCGTGGAGGCTGGTCTGTGCGCAAGCATGGAGCAGTTCTTGTGAAC

GCATTCCCTTTTTCATTTCTGGTCTCAAATCAGGTAGGATCACCCGCTGAACTTAAGCATATCAATAAGCCGGAGGAAAG

GATCATTAGTGAAAGCAAGGGCCAGCCATACGGACGGCGCTCCTCCGTACACGTCTCTGGCGCCAACTTTAACAATATCC

CAACCCGTGTGCCCGTTTGGAATGAATGGGGCAAGAGGCTCGGCTCTAATCTTTCTCCAACTCGTAAGGGTTGTATGAAA

CGTGGGAAATCTTTGGATCGTAACT

>CS_67_Malassezia_furfur

CCTAGCGGCACGAGGTCGTAGGTGGAGCTGCGGCAGCTCTCGTAAGCGGCGGAGGCGCCCAACTTTACACAATATCCACA

AACCCGTGTGCACCGTTTGGATGAGTTGGACCTCGCAAGAGGCCTGGCTCTCCAATCCATTTCTACCAAACTCGTATGGT

TGTATGAACGTGGAAATCGTTGGACCGTAACTGGCCAACAACCAATAATACAACTTTCGACAACGGATCTCTTGGTTCTC

CCATCGATGAAGAACGCAGCGAAACGCGATAGGTAATGTGAATTGCAGAATTCCGTGAATCATCGAATCTTTGAACGCAC

CTTGCGCTCCATGGTATTCCGTGGAGCATGCCTGTTTGAGTGCCGTGAATTCTCTCTCCCCAAGCGGTTGCGATTGCACT

GCTTTGGCGGACGAGGTTGGATGGGTGCTTCTGCCTGTTTCGCAAGAAACAGGCTCGCCCGAAATGCATTAGCGCCTTTG

GGACACACTCTGCAAACCGCTCTGAAAGGGAGGGCGGCAGAGGGGATGGAGGAACTCCGCCCGTCAGCTATACCAAACTT

TGCCCCAGGCAAGCGCATGGCATGATACGTCATTTGCTGTGTGTGCGTCCTGAGGCAAGGGGCCGATGTGGTGCCTTTGT

CACTCTGTGGGTGTGTTGGTGCGCTACCAATCCTGCGTGGAGGCTGGTCTGTGCGCAAGCATGGAGCAGTTCTTGTGAAC

GCATTCCCTTTTTTCATTTCTGGTCTCAAATCAGGTAGGATCACCCGCTGAACTTAAGCATATCAATAAGCCGGAGGAGA

AGGATCATTAGTGAAAGCAAGGGCCAGCCATACGGACGGCGCTCCTCCGTACACGTCTCTGCGCCCACTTTAACAATATC

ACAACCCGTGTGCACGTTTGGATGAGTTGGGACCTCCCAGGAGGCCTGGCTCCCATCATTTCACAAACTGTATGGGTTGT

ATGAAACGTGGAAAATCTTTGGACCGTAACTGGCCCACATAAATAACTTTC

>CS_68_Malassezia_furfur

GTGGCTGTGAGCAGGCCAGCCATACGGACGGCGCTACTCGCGTACAACGTCTCTGGCGCCCAACTTTACACAATATCCAC

AAACCCGTGTGCACCGTTTGGATGAGTTGGACCTCGCAAGAGGCTCGGCTCTCCAATCCATTTCTACCAAACTCGTATGG

TTTGTATGAACGTGGAAATCGTTGGACCGTAACTGGCCAACAACCAATAATACAACTTTCGACAACGGATCTCTTGGTTC

TCCCATCGATGAAGAACGCAGCGAAACGCGATAGGTAATGTGAATTGCAGAATTCCGTGAATCATCAAATCTTTGAACGC

ACCTTGCGCGCGATGGGATTCCCTGGAGCATGCCTGTTTGAGCGCCGAGAATTCTCTCTCCCCGAGCGGAGGCAAT

>CS_2-1_Malassezia_furfur

CCCTAGCGGCAGAGTCGTAGGAGGAGCATGGCGCTACTCGCGTACAACGTCTGAGGCGCCCAACTTTACACAATATCCAC

AAACCCGTGTGCACCGTTTGGATGAGTTGGGCAAGAGGCTCGGCTCTCCAATCCATTTCTACCAAACTCGTATGGTTGTA

TGAACGTGGAAATCGTTGGATCGTAACTGGCCAACAACCAATAATACAACTTTCGACAACGGATCTCTTGGTTCTCCCAT

CGATGAAGAACGCAGCGAAACGCGATAGGTAATGTGAATTGCAGAATTCCGTGAATCATCGAATCTTTGAACGCACCTTG

CGCTCCATGGTATTCCGTGGAGCATGCCTGTTTGAGTGCCGTGAATTCTCTCTCCCCAAGCGGTTGCGATTGCACTGCTT

TGGCGGACGAGGTTGGATGGGTGCTTCTGCCTGTTTCGCAAGAAACAGGCTCGCCCGAAATGCATTAGCGCCTTTGGGAC

ACACTCTGCAAACCGCTCTGAAAGGGAGGGCGGCAGAGGGGATGGAGGAACTCCGCCCGTCAGCTATACCAAACTTTGCC

CCAGGCAAGCGCATGGCATGATACGTCATTTGCTGTGTGTGCGTCCTGAGGCAAGGGGCCGATGTGGTGCCTTTGTCACT

CTGTGGGTGTGTTGGTGCGCTACCAATCCTGCGTGGAGGCTGGTCTGTGCGCAAGCATGGAGCAGTTCTTGTGAACGCAT

TCCCTTTTTCATTTCTGGTCTCAAATCAGGTAGGATCACCCGCTGAACTTAAGCATATCAATAAGCCGGAGGAAAGGATC

ATTAGTGAAAGCAAGGGCCAGCCATACGGACGGCGCTTCTCCGTACAACGTCTCTGGCGCCAACTTTACACAATATCCCA

AACCGTGTGCACGTTTGGAATGAAGTTGGGCAAGAGGCTCGGCTCTCATCCTTTTTCCAAATCCTAGGGTTGGTATGAAA

CTTGGGAAATCGTTGGGATCGTAACTGGGCCACAAAAATAAATAAACTTTTCAACGGAACCTTGGGGTTCCCTCATTAAA

AAGAACCCCCCAACCCCTTGGGGGTGAGGGGGAAATTTTCAA

>CS_72_Malassezia_restricta

GGGCTGGTGAGATTGCGGCAGGCCATACGGACGCCGAAGTGTCCCTGAGCCGCCTACACCCACTATACATCCACAAACCC

GTGTGCACTGTCTTGGAAAAAGGGTTCCTAGATTTTTTTGTGGCCTCTCTTGGGGTCTTTCTTCGCTACAAACTCGAATG

GTTAAATAAACGTGGAGTTGGTTGGACCGTCACTGGCCAATAAGGCTATCCACTACTTTCCACCAGGAATCTTTGGGGGT

CTCCCATCTAAAAAAAAACGCGGAAAAACCCTATAGGTTTTGTGAATTGCAAATTTCCGGGAATCCTCGAATCTTTGAACG

CACCTTGCCCTCTATGGAAATACGTAGGTATACATGTATGAATGCCTTGAAATCTCCCGCGCAAAACGGTTTTAGAATAT

GAAAAGGGATGCGCAGATGGAAAACAGGATAGATTG

>CS_74-4_Malassezia_japonica

AGTAGTAGGCGGAGTACGTCAGGAGGTGACCTGCAGGACGTATTATCTCTGGGAGGACGACCCTTTACACAAACATCCAA

ACCCGTGTGCACCTGTAGTCTGTTGGGCTTGAGCAATCGAGCCGGGCAGCTACGCAAATTTAAACCAAACTCGTATGGTT

GTATGAACGTGTAAATCGTTGGACCGTAACTGGCCAGCAAACAAACAATACAACTTTCGACAACGGATCTCTTGGTTCTC

CCATCGATGAAGAACGCAGCGAAACGCGATAGGTAATGTGAATTGCAGAATTCCGTGAATCATCGAATCTTTGAACGCAC

CTTGCGCTCCATGGTATTCCGTGGAGCATGCCTGTTTGAGTGCCGTGAATTCTCTCTCCCCAAGCGGTTGCGATTGCGCT

GCTTGGCGGATAGGTTGGATGGGTGCCTGCCAGTGTCTTGTACACCGGCTCGCCCGAAATTCATTAGCGCCTTGGACACA

CTCTGAAAACCGCCCCCGAAGGGAGGGCGGCAGAGCGGATGGAGGAATTCCACCCGTCGACTATACCAAACCTTGCCCAG

GCAAGCGCATGGCATGATACGTCATTTGCTGTGTGCGCGTGCTGGACCAAGGGGTTGATGCGGTGCCTCTAGAGCTTTGT

GGGTGTGTTGTGTGCTACGAACTATGCGTGGAGGCACTTGTGCCTTGTGAACGCGTTCCCTTTTTCATTTCTGGTCTCAA

ATCAGGTAGGATCACCCGCTGAACTTAAGCATATCAATAAGCGGAGGAAGATC

>CS_75-4_Malassezia_japonica

TATAGGCGGTGGTACGTCAGTAGGTGACCTGCTAGACGAGCTATCTCTGGGGCGACGACCTTTACACAAACATCCAAACC

CGTGTGCACCTGTAGTCTGTTGGGCTTGAGCAATCGAGCCGGGCAGCTACGCAAATTTAAACCAAACTCGTATGGTTGTA

TGAACGTGTAAATCGTTGGACCGTAACTGGCCAGCAAACAAACAATACAACTTTCGACAACGGATCTCTTGGTTCTCCCA

TCGATGAAGAACGCAGCGAAACGCGATAGGTAATGTGAATTGCAGAATTCCGTGAATCATCGAATCTTTGAACGCACCTT

GCGCTCCATGGTATTCCGTGGAGCATGCCTGTTTGAGTGCCGTGAATTCTCTCTCCCCAAGCGGTTGCGATTGCGCTGCT

TGGCGGATAGGTTGGATGGGTGCCTGCCAGTGTCTCGTACACCGGCTCGCCCGAAATTCATTAGCGCCTTGGACACACTC

TGAAAACCGCCCCCGAAGGGAGGGCGGCAGAGCGGATGGAGGAATTCCACCCGTCGACTATACCAAACCTTGCCCAGGCA

AGCGCATGGCATGATACGTCATTTGCTGTGTGCGCGTGCTGGGCCAAGGGGTTGATGCGGTGCCTCTAGAGCTTTGTGGG

TGTGTTGTGTGCTACGAACTATGCGTGGAGGCACTTGTGCCTTGTGAACGCGTTCCCTTTTTCATTTCTGGTCTCAAATC

AGGTAGGATCACCCGCTGAACTTAAGCATATCAATAAGCGGAGGAAGGATCT

>CS_78-4_Malassezia_furfur

TTGTGGGACGAGTACGTCCGTATTGTTGGCGCTGCTCACTCTCTCGTCTCTGGCGCCCTACTTTACATTCATCCACTTCC

CGTGTGGACGTTTGGTGAGTTGGGCAAGAGGCACGGCTCTCCAATGCAATTCTACCTAACACTCATGGCTGTATGAATCT

TCAAATCTTTGGACCCACATTGGCCATTGGGTATTCTACATGGTTTGATTGTTTATCCCATGACTTCCCCATCCATCACG

AATTGTATTGTGTTTGATCGGTAATGTTAATTACAGAATTCTACGAATCGGTACCTCTTTTAACCTCATTTTTTTTCCTC

GGATTTCCTAGGACTACCCCCTGAACTTTGCCATATCTTCTCTCGGAGGAAGCGGGTGCGATTGCACTGCTTTGGCGGAC

GAGGTTGGATGGGTGCTTCTGCCTGTTTCGCAAAAAACAGGCTCACCCGAAATGCATTAACGCCTTTGGGACACACTCTG

CAAACCGCTCTGAAAGGGAGGGCGGCACAGGGGATGGAGGAAATCCTCCCATCAGCTATACCAAACTTTGGCGCATGCCA

GCGCATGGCTTGATACATCAAATACTCCCTGTGCGTCCTGGAGCAAGGGGCCGATGAAAAATGATTTATAACTCTGTGGC

TGTGGGGCGCGCTACACAATCTTTTGCTCAGGGGTTGGGGAAGCCCGCAATAAAGGAGGCCCTCAAGGAGGAGAGAATAC

CACTTTTCATCTCTCGGGGTCAGGAGACGGCGTGGTGATCCCCTCTGGAGAACTTTCCCCCTCATGTAACACGCAGAAAG

AGAGGAATCATGTAGTGAAAGCCATGGCCAAGCCAATACGGACGGCGCCTACTACGCGTACAACGTCCTCTGGCGCCCAA

CTTTTACACAATAATCAACAAACCCCGTGTGCACCGTTTGGGAATGAAGTTTGGGCAAGAAGGCCTCGCTTCTCATCATT

TTTCTCTACAAAACTGCTTAGGGATTGTAATTGAAACCGTGGGAAAAATCGATTGGACCGGTAACCTGGCCCAACACAAT

TAAATATCAACCTTTTAACACTAAAACTTTTGGCTTTCTCGTCCAATTAAATAAAACGCAGCGAAACGCCAGTGAAGGCT

AAGTGGTAGAAATTGCGAAGAAGTGTCCGTGTAAAATCTGC

>CS_85_Malassezia_slooffiae

TGTAGTAGCGGCGGTAGTCAGGAGGTGCACCTGCGGAGGAATCATCAAGCGGAGGAGGAATTCATCCACAAACCCTGTGC

ATTGTACGGAGAAGAAGAGGTTGCGAATGGCGACCTATGGGTCTGCTTGTTTGTTGCCTCTTCTCTCCTTCTCTAAAAAC

ACGCATGTTTGTATGAACGTGATTGATGTTGGTTCACAACCAACAAACAAAACACAACTTTCGACAACGGATCTCTTGGT

TCTCCCATCGATGAAGAACGCAGCGAAACGCGATAGGTAATGTGAATTGCAGAATTCCGTGAATCATCGAATCTTTGAAC

GCACCTTGCGCTCTGTGGTATTCCGCAGAGCATGCCTGTTTGAGTGCCGTGAAATCTCTCCCCCCAAGTGGTGTTTGTAT

TAGGACGCTACGCGGCGGCGGAGGTTGGATGAGCGTCGTGCCTACTACTGGTAGGCTCGCTCGAAAAGCATGAGCGCTTG

AGAACACTTGTCGCCTGTGAGAGGCAGGCGGGCAGAACAGGTGAGCGAATGCTTCTCTGCGGAGAAGCGCCCACCGGTCT

GCGTGTGCGCATGGCATGATACGTTCCTTGCTATGTGTGGCCGCGACCAGTCTTGGGTTAGTAGCTTCTTGCTTCTGGCG

TGATCTCGTGTGCGCTTCCAATGGCACAGCCCTCTCTTCACTTCTGGCCTCAAATCAGGTAGGATCACCCGCTGAACTTA

AGCATATCAATAAGCGGAGGAAGAT

>CS_86-5_Malassezia_slooffiae

ATATAGGGCGGGCATGTAGGTCAGGAGGTGACCTGCGGAGGAATTGCCTCTCGGAGGAGCGACTATTTATCCACAAACCC

TGTGCACCGTGTGGAAGCGCGTTTGGATTGCTAATGGCGTCCGAGCGCCTTCCTTCTCTAAAAACACGCATGTTTGTATG

AACGTGATGGATGTTGGTTCACAACCAACGAACAAAACACAACTTTCGACAACGGATCTCTTGGTTCTCCCATCGATGAA

GAACGCAGCGAAACGCGATAGGTAATGTGAATTGCAGAATTCCGTGAATCATCGAATCTTTGAACGCACCTTGCGCTCTG

TGGTATTCCGCAGAGCATGCCTGTTTGAGTGCCGTGAAATCTCTCCCCCCAAGTGGTGTTTGTATAGGACGCTACGCGGC

GGCGGAGGTTGGATGAGCGTCGTGCCTACTACCGCGCTTGGTAGGCTCGCTCGAAAAGCATGAGCGCTTGAGAACACTTG

TCGCCTGTGAGAGGCAGGCGGGCAGAACAGGTGAGCGAATGCTTCTCCTCCGGAGAAGCGCCCACCGGTCTGCGTGTGCG

CATGGCATGATACGTTCCTTGCTATGTGTGGCGCGCAAACCCCATCTGGGTTAGTAGCTTCTTGCTTCTGGCGTGATCTC

GTGTGCGCTTCGAATCGCACAGCCCTCTCTTCACTTCTGGCCTCAAATCAGGTAGGATCACCCGCTGAACTTAAGCATAT

CAATAAGCCGGAGGAAAACGTCAGTGAGTTGCCTGGGGTTAGAAATACGCACGCTAACACGACGTGCCTCTCCAATCACT

ATTTATCACAAACCCTGTGCACCGTGTGGGAAGCGCGTTTGGAATTGCTAATGGGCGTCCAACCCTTCTTCTCTAAAAAA

CGCAGGTTTGTTATGGAAGATGAATGGGAAGGTTGGGTTCACACCCACGAAAAAAACAATTTTAAACGAATCTTGGGTTT

CCCCCTTTAAAAAAACCCCCACCCCCTCGGGTTTATGGGTAAATTGCAAAAAAATCCGCAAATCCCCAAACCTTTTAAAC

CCCCCCCCTGTGGGGATTTTCCCCAAAAAAGCCCCTTTTTAAAATGGGCGCCAACCCCCCCCGCCCCTTTTTTTTATTAT

AAACTCCCCCCCCCGCCCCAGGGGGGGAAAAATGACCTCCTTCCC

>CS_89-1_Malassezia_japonica

ATAGTAGGCGGAGTACGTCAGGAGGTCCAGCGGCGGAGTTATCAATAAGCGGAGGACGTCCGTTGGCACAAACATCCGAA

CCCGTGTGCACCTGTAGTCTGTTGGGCTTGAGCAATCGAGCCGGGCAGCTACGCAAATTTAAACCAAACTCGTATGGTTG

TATGAACGTGTAAATCGTTGGACCGTAACTGGCCAGCAAACAAACAATACAACTTTCGACAACGGATCTCTTGGTTCTCC

CATCGATGAAGAACGCAGCGAAACGCGATAGGTAATGTGAATTGCAGAATTCCGTGAATCATCGAATCTTTGAACGCACC

TTGCGCTCCATGGTATTCCGTGGAGCATGCCTGTTTGAGTGCCGTGAATTCTCTCTCCCCAAGCGGTTGCGATTGCGCTG

CTTGGCGGATAGGTTGGATGGGTGCCTGCCCGTGTCTTGTACACCGGCTCGCCCGAAATTCATTAGCGCCTTGGACACAC

TCTGAAAACCGCCCCCGAAGGGAGGGCGGCAGAGCGGATGGAGGAATTCCACCCGTCGACTATACCAAACCTTGCCCAGG

CAAGCGCATGGCATGATACGTCATTTGCTGTGTGCGCGTGCTGGACCAAGGGGTTGATGCGGTGCCTCTAGAGCTTTGTG

GGTGTGTTGTGTGCTACGAACTATGCGTGGAGGCACTTGTGCCTTGTGAACGCGTTCCCTTTTTCATTTCTGGTCTCAAA

TCAGGTAGGATCACCCGCTGAACTTAAGCATATCAATAAGCGGAGGAAGATC

>CS_90-2_Malassezia_arunalokei

GGTTTCCGGTGACAGTAGGTGCATGCCATACGGACGCCAAAAGTGTCCCTGGCCGCCTACACCCACTATACATCCACAAA

CCCGTGTGCACTGTCTTGGAGAAAGGCTTCTTGAGAAGTTTTGTGGCCTCTCTTGGGGTCTTTCTTCGCTACAAACTCGA

ATGGTTAGTATGAACGTGGAACTTGGTTGGACCGTCACTGGCCAACAAAACTATACACAACTTTCGACAACGGATCTCTT

GGTTCTCCCATCGATGAAGAACGCAGCGAAACGCGATAGGTAATGTGAATTGCAGAATTCCGTGAATCATCGAATCTTTG

AACGCACCTTGCGCTCTATGGCAATCCGTAGAGCATGCCTGTTTGAGTGCCATGAAATCTCCCACCCCAAGCGGTTTTTG

AATGTGAAACGGCTTGGCGGATGGGGTCTGGATGGGTGCCTCTGCCTGTGCTAAACCTTAGCTCACAGGCTCGCCCGAAA

TGCATGAGCGCCTTGGGACACTTTGCATCCGCCTCTCTTGTGGGAGGAGGCGGCCAAGCAGTGTTTTTCTCCAGGCATGG

CATGATACGTCATTTGCTATGTCGCCCAATGGAGGAATGTTTGGTTGTGTCTGCGTGTGCTTCGAACTTGCCTCTGTGCA

AAAATCCCCTTTCACTTCTGGTCTCAAATCAGGTAGGATCACCCGCTGAACTTAAGCATATCATAAGCCCGGAGGAAG

>CS_90-3_Malassezia_furfur

TGTAAGTAGGCAGAGTCGTCAGTAGGTGCTGGTGCTGCTCACATAAACGTCTGAGGAGCCCAACTTTACACAATATCCAC

AAACCCGTGTGCACCGTTTGGATGAGTTGGGCAAGAGGCTCGGCTCTCCAATCCATTTCTACCAAACTCGTATGGTTGTA

TGAACGTGGAAATCGTTGGACCGTAACTGGCCAACAACCAATAATACAACTTTCGACAACGGATCTCTTGGTTCTCCCAT

CGATGAAGAACGCAGCGAAACGCGATAGGTAATGTGAATTGCAGAATTCCGTGAATCATCGAATCTTTGAACGCACCTTG

CGCTCCATGGTATTCCGTGGAGCATGCCTGTTTGAGTGCCGTGAATTCTCTCTCCCCAAGCGGTTGCGATTGCACTGCTT

TGGCGGACGAGGTTGGATGGGTGCTTCTGCCTGTTTCGCAAGAAACAGGCTCGCCCGAAATGCATTAGCGCCTTTGGGAC

ACACTCTGCAAACCGCTCTGAAAGGGAGGGCGGCAGAGGGGATGGAGGAACTCCGCCCGTCAGCTATACCAAACTTTGCC

CCAGGCAAGCGCATGGCATGATACGTCATTTGCTGTGTGTGCGTCCTGAGGCAAGGGGCCGATGTGGTGCCTTTGTCACT

CTGTGGGTGTGTTGGTGCGCTACCAATCCTGCGTGGAGGCTGGTCTGTGCGCAAGCATGGAGCAGTTCTTGTGAACGCAT

TCCCTTTTTCATTTCTGGTCTCAAATCAGGTAGGATCACCCGCTGAACTTAAGCATATCAATAAGCCGGAAGGAAAGGAT

CCTTAGTGAAAGCAAGGGCCAGCCATACGGACGGCGCTTCTGGAAAAACGTCTCTGGCGCCAACTTTACACAATATCCCA

AACCCGTGTGCCCGTTTGGAATGAATTGGGGCAAGAAGGCTCGGCTCTCATCTTTTTACAAATCCTAGGGGTTGTTATGA

AACGTGGAAAATCGTTTGGAACGGTAACTGGGCCACACAATAAAATAAATTGTCAGGGAAACTTGGGGTTTCCCCCATTA

AAAAAAACACCAAACCCTTTGGGTTTAAGGGGGGAATTGCCAAAAATTCCGAGAAATCCCCAAAATCTTTTTAAAC

>CS_195_Malassezia_sympodialis

GTTGCATCGATGAAGAACGCAGCGAAACGCGATAGGTAATGTGAATTGCAGAATTCCGTGAATCATCGAGTCTTTGAACG

CACCTTGCGCTCCATGGTATTCCGTGGAGCATGCCTGTTTGAGTGCCGCGAATTCTCCCTCCCCATACGGTGGCCGAAAG

GCCGGAGTATGGCGGACGGGGTTGGATGGGTGCCGCTGCCTGGGAAACCAGGCTCGCCCGAAATGCATAAGCGCCAGGAC

CCTCGCTACTGCCCTCTAGGGAAGGGCGGCTGAGCGACCGCTGAGCATGGCATGATACGTCATTTGCTGTGTGTGGGCGG

CCGGTTGGAGAGGTGTCTGCTTTATACAGCCCTTCCTTATTCTTGGTTCTACAAATTCATGGCCAGGCACACCCC

>CS_209-1_Malassezia_arunalokei

TTTTTCTGCATCGATGAAGAACGCAGCGAAACGCGATAGGTAATGTGAATTGCAGAATTCCGTGAATCATCGAATCTTTG

AACGCACCTTGCGCTCTATGGCAATCCGTAGGGCATGCCTGTTTGAGTGCCATGAAATCTCCCACCCCAAGCGGTTTTTG

AATGAAACGGCTTGGCGGATGGGGTCTGGATGGGTGCCTCTGCCTGTGCTTACCTTTTAGCCCACAGGCTCGCCCGAAAT

GCATGAGCGCCTTGGGACACTTTGCATCCGCCTCTCTTGTGGGAGGAGGCGGCCAAGCAGTGTTTTTCTCCAGGCATGGC

ATGATACGTCATTTGCTATGTCGCCCAATGGAGGAATGTTTGGTTGTGTCTGCGTGTGCTTCGAACTTGCCTCTGTGCAA

ATCCCCTTTCACTTCTGGTCTCAAATCAGGTAGTCCCCACCC

>CS_215-1_Malassezia_sympodialis

TTTGCATCGATGAAGAACGCAGCGAAACGCGATAGGTAATGTGAATTGCAGAATTCCGTGAATCATCGAATCTTTGAACG

CACCTTGCGCTCCATGGTATTCCGTGGAGCATGCCTGTTTGAGTGCCGCGAATTCTCCCTCCCCTTACGGTGGCCGAAAG

GCCGAAGTAGGGCGGACGGGGTAGGATGGGTGTTTGCTGCCTGGGGATTGTACCAGGCTCGCCCGAAATGCATAAGCGCC

AGGACCCTCGCTACCGCTCTCTAGGGAAGAGTGGCTAAGCGACCGCTGAGCATGGCATGATACGTCATTTGCTGTGTGTG

GGCGGCCGGTTGGAGAGGTGTCTGCTTTACCAGCCCTATTTTAATTCTGGTCTCAAATTCAGGTAGGTCCCCCCCC

>CS_219-1_Malassezia_slooffiae

CTGACCCACCTCCCCCAAACACCAAAGAGGTAGACACATACCCTCGCCCCCTTCCTGCATCGATGAAGAACGCAGCGAAA

CGCGATAGGTAATGTGAATTGCAGAATTCCGTGAATCATCGAATCTTTGAACGCACCTTGCGCTCTGTGGTATTCCGCAG

AGCATGCCTGTTTGAGTGCCGTGAAATCTCTCCCCCCAAGTGGTGTTTGTATAGGACGCTACGCGGCGGCGGAGGTTGGA

TGAGCGTCGTGCCTACCGCGCTTGGTAGGCTCGCTCGAAAAGCATGAGCGCTTGAGAACACTTGTCGCCTGTGAGAGGCA

GGCGGGCAGAACAGGTGAGCGAATGCTTCTCCTCCGGAGAAGCGCCCACCGGTCTGCGTGTGCGCATGGCATGATACGTT

CCTTGCTATGTGTGGCGCGCAAACCCCATCTGGGTTAGTAGCTTCTTGCTTCTGGCGTGATCTCGTGTGCGCTTCGAATC

GCACAGCCCTCTCTTCACTTCTGGCCTCAAATCAGGTGGATCCCCATCCCC

>CS_220-2_Malassezia_globosa

AGAGTTGGGCAAAACCCACCCTGGTGGGTGGCCCGGAATTTTCCACCCCACACGGGTTTTTTTTAAAAAATTTTTTGGGC

GGGGGGTTGGAGGGGGGGTTTTTTATAACCTTTTTTTTTTCTTGAAAAAAGCTCCCGGAAAAAAAAAGAAAAGCTTTGGGGC

CCCTTTGGCATCCTTTTTTGAGGGGGGAAAAGGGCCAAACCCCTTTGAACACTTCTCCGCTCCCAAAAAAGCCAAAATTC

AGCCGGTGGGGCGCCCCTGTTTAGAGCCAAAAAGAAGGCGGCCCTTTTTTTTTCTTGCAAAGGCGAAAAGAAAAAGCCCC

CCTTCATTCTGGTCTCAAATCAGGTAGATCTCCCAATCCCCC

>CS_107_Malassezia_globosa

CATACAGACGCTGCGATCCCTTTATGCTATTTTTTGGAGCCTGCGCAGGGACTGTAGGATCATCTCTTATTTTCTTAGTG

CCACCTTCCCTAAGTCAACCAGGGGCTTTTTTTTTTGAGGGATCCTCCGGTTAATGAAATTGTGGCTTTTTTAATGGGGC

CTCCCCCCCTTTATGGATTGCCGCGTTTTTTTAAGGGGGGGTCCCCCCGTTAAAGAACGCAGCATTTCTTTAAGCGGCGG

TCCTGCATCGATGAAGAACGCAGCGAAACGCGATAGGTAATGTGAATTGCAGAATTCCGTGAATCATCGAATCTTTGAAC

GCACCTTGCGCTCTATGGTATTCCGTAGAGCATGCCTGTTTGAGTGCCGTGAATTCTCCCATCCCAAGCGGTTTTTATCA

AAGAATTGCTAGGCGAAGGGGTTGAGATGGGTGCTGACTGCTTTTCTTCTCTAGAAAAGCTCACCCGAAAAACAGCTAAC

GCCTCTGGGCCACTTTGCATCCGCTTCTCTGAGGGGAGAAGCGGCCAAGCGCGCTCTGATCATCAGGCATAGCATGATAC

GTCATTTGCTATGCTGTAGGAGAGCATTGGGTCCTTGGATAACGAGCGCGGCAATTCCAATAAGGGGGGAATGCATGGAT

GAAGATCCGCAGCATTTCAATAAGGCGGAAGGAAGCTCGGAGCCCC

>CS_112_Malassezia_japonica

CTTTTGTGAATCCCTGCGCTTCTTTCCTTGCAGGAATTCTTTTCGTATTTATAAAATAAGGCGGTTTCTTTTTGGGAGGG

TCTCTCCCGTTTATGGATAAGGGGGGTTTTTTTTGGGGGGCTTCCTTCCTTTATGGAATGGGGGGGTTTTTTATGGAGGG

TTTCCCCCCTTAATGAATTGCGGGTTTTTTTAAGGGGGGGTTCTTCCGTTTAAGGATGGCAGCGTTTTTTTAAGGGGGGG

CTGCATCGTTGAAGAACGCAGCATTTCATTAAGCGGGGTTCCTTCCATCGATGAAGAACGCAGCGAAACGCGATAGGTAA

TGTGAATTGCAGAATTCCGTGAATCATCGAATCTTTGAACGCACCTTGCGCTCCATGGTATTCCGTGGAGCATGCCTGTT

TGAGTGCCGTGAATTCTCTCTCCCCAAGCGGTTGCGATTGCGCTGCTTGGCGGATAGGTTGGATGGGTGCCTGCCAGTGT

CTCGTACACCGGCTCGCCCGAAATTCATTAGCGCCTTGGACACACTCTGAAAACCGCCCCCGAAGGGAGGGCGGCAGAGC

GGATGGAGGAATTCCACCCGTCGACTATACCAAACCTTGCCCAGGCAAGCGCATGGCATGATACGTCATTTGCTGTGTGC

GCGTGCTGGGCCAAGGGGTTGATGCGGTGCCTCTAGAGCTTTGAGGGGGTGTTGTGTGCTACGAACTATGCGAGGAGGCA

ATTAAGCGGGGGATGCATGGATGAAGATTCGCAGCATTTCAATAAGGCGGAGAAGGCTCGAAGCCCC

>CS_138_Malassezia_restricta

CCCGCCCATTGATGCTTCCCGCATTTTTTACTTACCCAATCGCTTTCAATTACACATCCTGGGGGTTCTAATGCAGGCCT

CCCCCCCTTATTGATATGCTGGTTTTTTTATTGACGGATCCTCCCCTTTATTAATATGTCGGTTTCTTATTAAGGGGTGC

TTCCGTTGATGAAGATGGCGCCTTATCATTAAGCGGCTGCTTATGCATCGATGAAGAACGCAGCGAAACGCGATAGGTAA

TGTGAATTGCAGAATTCCGTGAATCATCGAATCTTTGAACGCACCTTGCGCTCTATGGCAATCCGTAGAGCATGCCTGTT

TGAGTGCCATGAAATCTCCCACCCCAAGCGGTTTTTAAATGAAACGGCTTGGCGGATGGGGTCTGGATGGGTGCCTCTGC

CTGCGCTACCTAGCACAGGCTCGCCCGAAATGCATGAGCGCCTGAGACACTTTGCATCCGCCTCTCTGTTTGGGAGGAGG

CGGCCAAGCAGTGTTTTTCTCCTGGCATGGCATGATACGTCATTTGCTATGTCGCCTAAAGGAGGAATGTTTGGTTGAGT

ATGCGAGCCATTCAAAAAAGCGGGGGTTGCACCGATGAAGATACGCAAGGCATATCAATAAGGCGGAGGGTGGCTCGAAG

CCCC

>CS_148-2_Malassezia_japonica

TGCCCTAAGCCTCTCCCTCCCTCACTTACATCTCCGCTCCTTCTCTTCTAGTTCCTTCTTGTCTATGTCAACATGCTGGG

TTTTTTATGGGAGGATTTTTCCTGTTTTTTGGTATGTTGGTTTTTTATTGGGAGCATTCTTGGCTTATGGAATTGGGGGG

TTTTTTATGGAGGGATCCCCCGGTTTATGAATTTCGGGTTTTTTTAAGGGGGGGTTCCTCCGTTTAAGGATCGCAGCATT

TCTTTAAGGGGGGGTTGCATCGTTGAAGAACGCAGCATTTCATTAAGCGGGGGTCCTGCATCGATGGAGAACGCAGCGAA

ACGCGATAGGTAATGTGAATTGCAGAATTCCGTGAATCATCGAATCTTTGAACGCACCTTGCGCTCCATGGTATTCCGTG

GAGCATGCCTGTTTGAGTGCCGTGAATTCTCTCTCCCCAAGCGGTTGCGATTGCGCTGCTTGGCGGATAGGTTGGATGGG

TGCCTGCCAGTGTCTCGTACACCGGCTCGCCCGAAATTCATTAGCGCCTTGGACACACTCTGAAAACCGCCCCCGAAGGG

AGGGCGGCAGAGCGGATGGAGGAATTCCACCCGTCGACTATACCAAACCTTGCCCAGGCAAGCGCATGGCATGATACGTC

ATTTGCTGTGTGCGCGTGCTGGGCCAAGGGGTTGATGCGGTGCCTCTAGAGCTTTGTGGGTGTGTTGTGTGCTACGAACT

ATGCGTGGAGGCACTTGTGCGTTAGGATGCGCGTTCCATTGATTCACATCTTGGTCAACAAAGCGGGGGGGTCCCCACGC

CCC

>CS_167-2_Malassezia_sympodialis

GTTTTGCATCGATGAAGAACGCAGCGAAACGCGATAGGTAATGTGAATTGCAGAATTCCGTGAATCATCGAGTCTTTGAA

CGCACCTTGCGCTCCATGGTATTCCGTGGAGCATGCCTGTTTGAGTGCCGCGAATTCTCCCTCCCCATACGGTGGCCGAA

AGGCCGGAGTATGGCGGACGGGGTTGGATGGGTGCCGCTGCCTGGGAAACCAGGCTCGCCCGAAATGCATAAGCGCCAGG

ACCCTCGCTACTGCCCTCTAGGGAAGGGCGGCTGAGCGACCGCTGAGCATGGCATGATACGTCATTTGCTGTGTGTGGGC

GGCCGGTTGGAGAGGTGTCTGCTTTATACAGCCCTTTTTATTCTGGTCTTCTAGCATCCAGGAGTGAGTTGCTCCGAGCC

CCC

>CS_169-2_Malassezia_japonica

CACTCCCAGCTCTTCCTCAGACCATCCTCCCGGTCCATCACTTCATAAAGATTCTAGAACCTTATTCTCTGCTTTCGGAA

CCTGCCCCTTTATAATTGAAGCCTTCTTCCGGCTTCTGAAGACTCTCCCTTTTTATAGGGGGGCGTTGTTCCTGTTTAAG

GATGGCAGCCTTTCTTAAAGGGGGGTTTCCACCGTTGAAGAACGCAGCATATCATTAAGCGGGGGTCCTGCATCGATGAA

GAACGCAGCGAAACGCGATAGGTAATGTGAATTGCAGAATTCCGTGAATCATCGAATCTTTGAACGCACCTTGCGCTCCA

TGGTATTCCGTGGAGCATGCCTGTTTGAGTGCCGTGAATTCTCTCTCCCCAAGCGGTTGCGATTGCGCTGCTTGGCGGAT

AGGTTGGATGGGTGCCTGCCAGTGTCTCGTACACCGGCTCGCCCGAAATTCATTAGCGCCTTGGACACACTCTGAAAACC

GCCCCCGAAGGGAGGGCGGCAGAGCGGATGGAGGAATTCCACCCGTCGACTATACCAAACCTTGCCCAGGCAAGCGCATG

GCATGATACGTCATTTGCTGTGTGCGCGTGCTGGGCCAAGGGGTTGATGCGGTGCCTCTAGAGCTTTGTGGGTGTGTTGT

GTGCTACGAACTATGCGTGGAGGCAATTGTGCGTGGGATGCGCGTTCCAAGATTCGCATCCTTATCAATAAGGCGGGGAT

GCACCAAGCCCC

>CS_179-1_Malassezia_japonica

GTCTTTACATACTATAGCCACTTAGACGAATAATGGTTTTAACTATTGATGAAAATGATAGTTTTTTTTATTTAGAGATT

CTTCGCGTTAAAGGATTGGCGGCTTTTTTTTTATAGGGGGGGTTGCTTCGTTGAAGAACGCAGCATTTCATTAAGCGGCG

GTTTCTGCATCGATGAAGAACGCAGCGAAACGCGATAGGTAATGTGAATTGCAGAATTCCGTGAATCATCGAATCTTTGA

ACGCACCTTGCGCTCCATGGTATTCCGTGGAGCATGCCTGTTTGAGTGCCGTGAATTCTCTCTCCCCAAGCGGTTGCGAT

TGCGCTGCTTGGCGGATAGGTTGGATGGGTGCCTGCCAGTGTCTTGTACACCGGCTCGCCCGAAATTCATTAGCGCCTTG

GACACACTCTGAAAACCGCCCCCGAAGGGAGGGCGGCAGAGCGGATGGAGGAATTCCACCCGTCGACTATACCAAACCTT

GCCCAGGCAAGCGCATGGCATGATACGTCATTTGCTGTGTGCGCGTGCTGGACCAAGGGGTTGATGCGGTGCCTCTAGAG

CTTTGTGGGTGTGTTGTGTGCTACGAACTATGCGTGGAGGCAATTATGCGGGGGATGCATGGATGAAGTACTCCGCCATG

CATATCCATTAAAGGCGGAGGTTGGCTCGAAGCCCC

>CS_180-2_Malassezia_furfur

CCTCCTCTTTTAAAATCCGGGGTTTCTCTTAGAAGGATTCCTCGCTTAATGAATAGGCGGGTTTTTTTATGGGGGAGTTC

TTCGGTTATTGGAATTGGGGGTTTTTTTTTGGGGGCATTCTCCCTTTATAAAATTGCCGGCTTTTTATTAGGGGGGGCCT

CCCTCTATGAAAATGGCGCCTTTTTAATAAGGGGAGTCTGCATCGATGAAGAACGCAGCATTTCAATAAGCGGCGTCCTC

TGCATCGATGAAGAACGCAGCGAAACGCGATAGGTAATGTGAATTGCAGAATTCCGTGAATCATCGAATCTTTGAACGCA

CCTTGCGCTCCATGGTATTCCGTGGAGCATGCCTGTTTGAGTGCCGTGAATTCTCTCTCCCCAAGCGGTTGCGATTGCAC

TGCTTTGGCGGACGAGGTTGGATGGGTGCTTCTGCCTGTTTCGCAAGAAACAGGCTCGCCCGAAATGCATTAGCGCCTTT

GGGACACACTCTGCAAACCGCTCTGAAAGGGAGGGCGGCAGAGGGGATGGAGGAACTCCGCCCGTCAGCTATACCAAACT

TTGCCCCAGGCAAGCGCATGGCATGATACGTCATTTGCTGTGTGTGCGTCCTGAGGCAAGGGGCCGATGTGGTGCCTTTG

TCACTCTGTGGGTGTGTTGGTGCGCTAACAATCGGGGGTGGAGGCTGGTGAGGGCGCAAGCATGCCAACAAATCGGGGAA

TCCCTCGATGAAGATTCCGCAGCATATCCAATAAGGCGGGGGAAGGCATCGAAGCCCC
